# Spotlight on iron and ferroptosis: research progress in diabetic retinopathy

**DOI:** 10.3389/fendo.2023.1234824

**Published:** 2023-09-13

**Authors:** Junlin Ouyang, Ling Zhou, Qing Wang

**Affiliations:** ^1^ Department of Endocrinology, China–Japan Union Hospital of Jilin University, Changchun, Jilin, China; ^2^ Department of Obstetrics and Gynecology, China–Japan Union Hospital of Jilin University, Changchun, Jilin, China

**Keywords:** iron, ferroptosis, cell death, mechanism, diabetic retinopathy

## Abstract

Iron, as the most abundant metallic element within the human organism, is an indispensable ion for sustaining life and assumes a pivotal role in governing glucose and lipid metabolism, along with orchestrating inflammatory responses. The presence of diabetes mellitus (DM) can induce aberrant iron accumulation within the corporeal system. Consequentially, iron overload precipitates a sequence of important adversities, subsequently setting in motion a domino effect wherein ferroptosis emerges as the utmost pernicious outcome. Ferroptosis, an emerging variant of non-apoptotic regulated cell death, operates independently of caspases and GSDMD. It distinguishes itself from alternative forms of controlled cell death through distinctive morphological and biochemical attributes. Its principal hallmark resides in the pathological accrual of intracellular iron and the concomitant generation of iron-driven lipid peroxides. Diabetic retinopathy (DR), established as the predominant cause of adult blindness, wields profound influence over the well-being and psychosocial strain experienced by afflicted individuals. Presently, an abundance of research endeavors has ascertained the pervasive engagement of iron and ferroptosis in the microangiopathy inherent to DR. Evidently, judicious management of iron overload and ferroptosis in the early stages of DR bears the potential to considerably decelerate disease progression. Within this discourse, we undertake a comprehensive exploration of the regulatory mechanisms governing iron homeostasis and ferroptosis. Furthermore, we expound upon the subsequent detriments induced by their dysregulation. Concurrently, we elucidate the intricate interplay linking iron overload, ferroptosis, and DR. Delving deeper, we engage in a comprehensive deliberation regarding strategies to modulate their influence, thereby effecting prospective interventions in the trajectory of DR’s advancement or employing them as therapeutic modalities.

## 1 Introduction

Globally, recent findings from the International Diabetes Federation reveal an impending surge in the population of individuals aged 20–79 afflicted by diabetes, projecting a staggering escalation to 642 million by the year 2040 (1). Concurrently, the incidence and prevalence of diabetes are on a steadfast ascent. In tandem, epidemiological investigations have proffered insights, indicating the presence of at least one complication in patients diagnosed with type 2 diabetes mellitus (T2DM), and a minimum of three complications in those afflicted by type 1 diabetes mellitus (T1DM) (2–4). Amidst this myriad of complications, the onset of diabetic retinopathy (DR) often requires a span of approximately one decade post-diabetes diagnosis for clinical manifestation, with an incidence ranging between 24.7% and 35.7% (5). DR, esteemed as the paramount catalyst for adult blindness, engenders a profound deterioration in both quality of life and psychological well-being, accounting for a substantial 22.27% of the cumulative diabetes-related burden (6). With the anticipation that the cohort of individuals grappling with diverse gradations of DR will reach a zenith of 120 million by 2025 (7), the imperative to address DR has heightened alongside the imperative of glycemic management. In the current landscape, a panoply of treatments, encompassing anti-vascular agents, laser photocoagulation, and surgical modalities, endeavor to arrest the progression of DR. However, their efficacy remains conspicuously suboptimal. Henceforth, an imperative endures to embark upon an exhaustive exploration into the intricate nosogenesis of DR, unfurling avenues that foster innovative paradigms for forthcoming clinical interventions.

Currently, the consensus pervades that DR represents an ocular malady characterized by aberrations within the microvascular and neural frameworks. Prominently, the pathological spectrum encompasses non-proliferative and proliferative manifestations (8, 9), all of which commence with the dysfunction of the blood-retina barrier (10, 11). The cardinal facets of microangiopathy encompass the perturbation of endothelial cells and pericyte architecture, while neuropathy predominantly culminates in neuronal demise (8, 9). Non-proliferative diabetic retinopathy (NPDR) chiefly materializes through escalated vascular permeability, engendering the ingress of substantial volumes of fluid, lipid peroxides, and an array of inflammatory mediators into the retinal milieu, thereby provoking macular edema. However, the incipient occurrence of neuronal impairments remains a subject of contention. In juxtaposition, the escalation of vascular permeability emerges as a pivotal hallmark in DR progression, a consequence of augmented permeability within retinal capillary endothelial cells and intercellular junctional disruptions (12). On the contrary, proliferative diabetic retinopathy (PDR) pivots upon pathogenic vascular proliferation. Yet, this phenomenon renders the retina susceptible to hemorrhage and, consequentially, detachment, owing to the heightened fragility intrinsic to neovascularization. Furthermore, following the occurrence of retinal detachment, a mere 40% of patients manage to achieve a visual acuity exceeding 20/40 within clinical settings (13).

**Figure 1:**
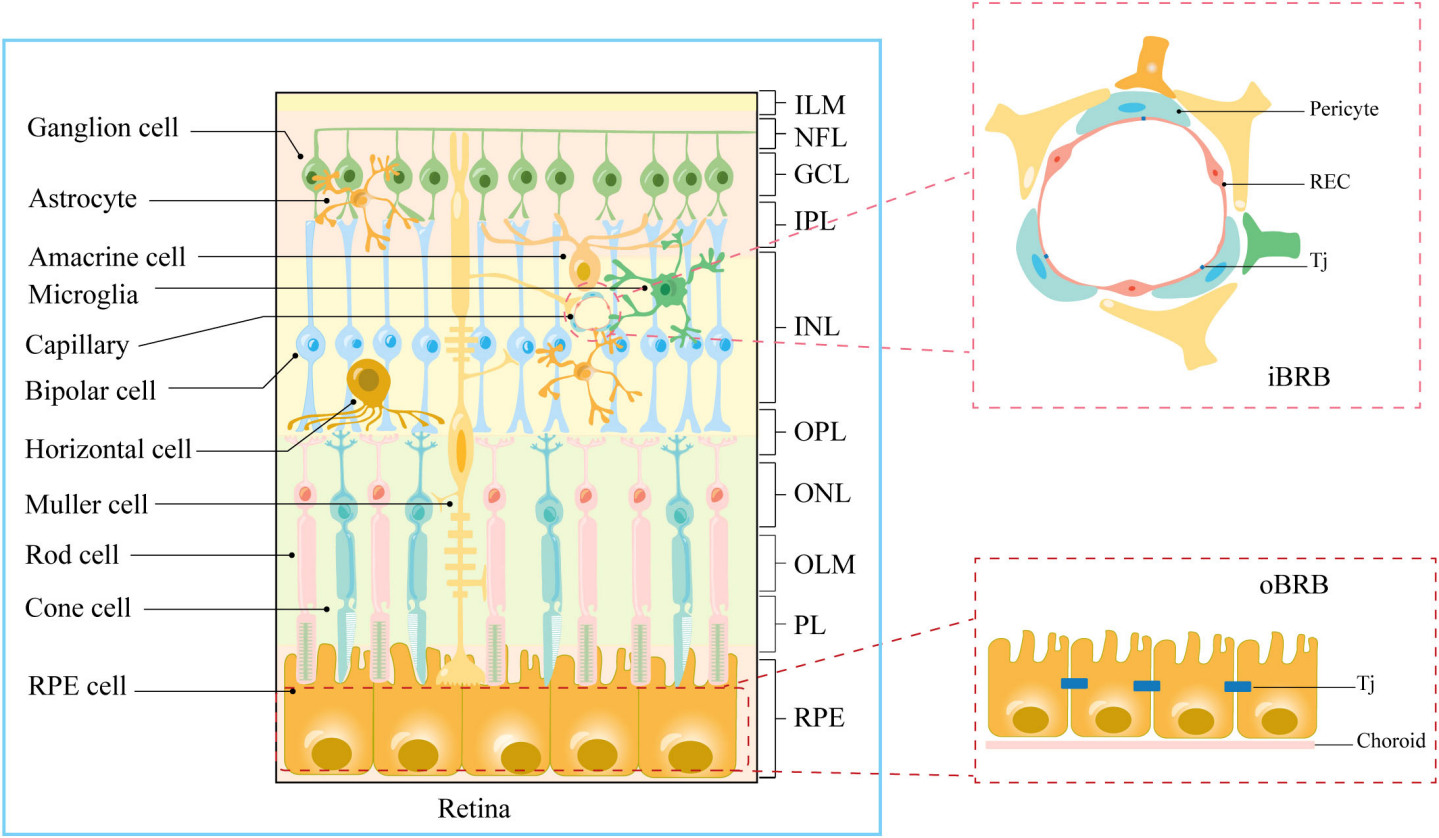
Anatomical stratification of retinal structures. Cellular and molecular structural components of iBRB and oBRB. ILM, internal limiting membrane; NFL, nerve fiber layer; GCL, ganglion cell layer; IPL, inner plexiform layer; INL, inner nuclear layer; OPL, outer plexiform layer; ONL, outer nuclear layer; OLM, outer limiting membrane; PL, photoreceptor layer; RPE, retinal pigment epithelium; Tj, tight junction.

The human retina, an intricate layer of photosensitive tissue nestled within the eye, constitutes a significant convergence of nerves and blood vessels (13). It stands as a pivotal constituent of the central nervous system, characterized by a bifurcated physiological and anatomical framework encompassing the neuro-retina and the retinal pigment epithelium (8) (**Figure 1**). Concomitantly, the retina is endowed with elevated concentrations of polyunsaturated fatty acids, a salient attribute resonating profoundly within macular photoreceptors, rendering them remarkably predisposed to peroxidative challenges posed by reactive oxygen species (ROS) (14, 15). Permeated by an inherent state of heightened metabolic activity, the retina registers as the most oxygen-consuming tissue per unit mass within the human anatomy (16). Furthermore, the influence of light stands manifest, stimulating robust ROS production within photoreceptors, thus bequeathing a hyperoxic milieu (8, 11). Ergo, the convergence of retinal structural attributes and the photo-oxidative milieu render it conspicuously susceptible to oxidative stress and inflammatory reactions elicited by ROS, thereby yielding photoreceptor degradation and ensuing visual impairments (14). Hence, safeguarding the microenvironment of the retina necessitates an imperative isolation from systemic circulation coupled with stringent defense mechanisms. Mirroring the paradigm of the blood-brain barrier, the blood-retina barrier (BRB) assumes a pivotal role in shielding the retina from systemic inflammatory elements and fluctuations in plasma composition (8, 9) (**Figure 1**). A bulwark for retinal integrity, the BRB is discernibly partitioned into the outer blood-retinal barrier (oBRB) and the inner blood-retinal barrier (iBRB). The oBRB coordinates the orchestration of nutrient-waste exchange, clearance of ROS, and oversight of inflammatory cell migration within the outer retinal domains, predominantly encompassing the choroid, Bruch’s membrane (BM), and retinal pigment epithelium (RPE) (8). Moreover, the melanin harbored by the RPE adeptly captures iron ions, effectively shielding the retina against oxidative damage (14, 17). Conversely, the iBRB emerges as an amalgamation of the neurovascular unit (NVU), an architectonic ensemble instrumental in regulating the stability of the retinal microenvironment (9).

The surfeit of advanced glycosylation end products, augmented expression of inflammatory cytokines, elevated polyol pathway activity, and heightened glucose concentrations, all stemming from the protracted hyperglycemic milieu, collectively wield the potential to incite cellular oxidative stress, inflammatory upheavals, mitochondrial compromise, and endoplasmic reticulum strain (8, 12, 18–21). Additionally, the shadow of insulin resistance further compounds endothelial cell dysfunction (22–24). These aforementioned adversities, akin to deleterious agents, progressively erode the structural integrity of the blood-retina barrier (BRB) (25), thus dismantling its protective shield over the retina. Consequently, the genesis or exacerbation of DR becomes an inevitable outcome. Notably, recent studies have illuminated the pivotal role of iron in the pathological underpinnings of DR (5, 6, 9, 10), with a pronounced emphasis on ferrous ions (26).

Iron, a trace element of paramount significance, assumes the dual role of an essential cofactor in critical physiological functions and numerous enzymatic processes, thereby exerting a distinct and influential sway over multiple metabolic pathways and regulatory circuits (6). The elucidation by Simcox et al. resolutely underscores the epidemiological nexus between diabetes and iron, establishing a causal association with unwavering clarity (18). Recent strides in the field, manifesting in a prospective cohort study (27), corroborate the inseparable connection between total and non-heme iron intake and the peril of diabetes. This dichotomy is emblematic: optimal intake of non-heme or total iron can ostensibly confer a protective shield against diabetes, while an excess of iron portends heightened susceptibility to its onset. The role of iron reverberates, with equal resonance, in the landscape of diabetic complications (28). Within the realm of DR, the onset of retinal damage is inextricably entwined with burgeoning oxidative stress, in which iron overabundance functions as both a catalyst and a co-factor in orchestrating oxidative upheaval. Remarkably, as human retinal iron content incrementally burgeons with advancing age, the imposition of diabetes itself precipitates pathological iron deposition within the retinal domain (10, 29). The influx of consumable iron within the retina triggers a cascade of hydroxyl radical generation through the Fenton/Haber–Weiss reaction, culminating in an escalation of oxidative stress that, in turn, propagates lipid peroxidation and inflicts damage upon retinal pigment epithelial cells (RPECs), retinal endothelial cells (RECs), and neurons, potentially catalyzing the instigation of ferroptosis. Beyond Fe^2+^, the orchestration of the Fenton reaction necessitates the intervention of hydrogen peroxide. The metabolic distortions ensuing from diabetes unleash oxidative stress, triggering the transformation of xanthine dehydrogenase (XDH) into xanthine oxidase (XO), precipitating hydrogen peroxide generation via two-electron reduction of O_2_ (26). Moreover, the high-glucose milieu and escalated inflammation levels act as direct instigators of ferroptosis (30). Notably, the distribution of iron within retinal cells is profoundly heterogeneous, especially reigning preeminent within the retinal pigment epithelium and the inner segments of photoreceptors (31). Consequently, the extent of damage wrought by iron surplus manifests as a variable spectrum across different cellular constituents. Although the aftermath of ferroptosis uniformly echoes in diverse cell types, the intricacies of the molecular underpinnings deviate extensively. Evidently, comprehending the intricate molecular tapestry of iron overload in the context of DR stands as an imperative, engendering the exploration of potential therapeutic targets.

Within this discourse, we unfurl the details of the intricate regulatory apparatus governing retinal iron homeostasis, unveiling both direct and indirect lines of evidence substantiating iron’s role in instigating and perpetuating the trajectory of diabetic retinopathy.

## 2 Iron and its biological effects

Undoubtedly, iron stands as the preeminent metallic constituent within the human corporeal framework, intricately woven into the synthesis of heme and iron-sulfur (Fe-S) clusters within the mitochondrial matrix. This elemental foundation serves as a cornerstone for a multitude of enzymes and proteins that choreograph a symphony of cellular metabolisms and physiological functions. Among these orchestrators are hemoglobin, ferredoxin, nitric oxide synthase, succinate dehydrogenase, cytochrome c oxidase, cytochrome P450s, xeroderma pigmentosum group D (XPD), and DNA polymerase, each delineating a distinct role (6, 17, 18). The canvas broadens still further, with iron assuming a pivotal role in steering the production of red blood cells within the hematopoietic system, encompassing a far-reaching array of influences. Research affirms iron’s indispensability in the growth and maturation of immune cells, particularly neutrophils, thus orchestrating a finely tuned regulation of the human immune apparatus via their modulation (32, 33). Intricately interlacing its influence through this network of proteins and enzymes, iron emerges as an unequivocal orchestrator in manifold cellular processes that span the spectrum from physiological to pathological. The gamut traversed encompasses fuel oxidation, cellular kinetics, oxygen conveyance and storage, mitochondria-tRNA modification, DNA synthesis and mending, as well as the inactivation of pharmaceutical agents and noxious agents (17). Enzymes furnish a pivotal “crowbar” for the oxidative metabolism of diverse substances, while mitochondria proffer a steady fulcrum upon which this metabolic machinery pivots. In a reciprocating dance, iron undertakes the critical mantle of governing mitochondrial DNA transcription, bolstering the stability of the mitochondrial network, galvanizing mitochondrial biogenesis, and amplifying the synthesis of adenosine triphosphate (13, 34, 35). In the intricate choreography of intracellular dynamics, it is through this triad of mitochondria, enzymes, and proteins that intracellular iron emerges as a mediating conduit, steering the regulation of organismal substance metabolism. In essence, the web of connections woven by iron permeates the fabric of human biology, manifesting its influence through the intricate interplay of mitochondria, enzymes, and proteins, thereby governing the multifaceted tapestry of substance metabolism within the organism.

Iron assumes an indispensable mantle within retinal physiology, occupying a nonpareil position. Of noteworthy mention is neuroglobulin (Ngb), which stands as an abundantly expressed constituent within the retinal milieu. As a fresh entrant to the hemoglobin family, Ngb exerts a reversible binding affinity for oxygen, orchestrating the transfer of oxygen from the circulatory blood to neurons. This symphony serves the imperious purpose of satiating the oxygen demands of the retina, endowed with its energy-intensive disposition (21). Commensurate with its pivotal role, the retinal pigment epithelium 65 kDa protein emerges as an iron-laden isomeric hydrolase. This formidable entity undertakes the hydrolysis of all-trans retinal ester into 11-cis retinal ester, known as the light-absorbing chromophore. This constituent ascends to the vanguard as an elemental component of rhodopsin, a centerpiece within the visual cycle (17). It bears profound significance, pivotal for the fidelity of vision. Egregious anomalies in all-trans retinal ester accumulation within the retina can precipitate drastic plummeting of photoreceptor cell activity, a perilous trajectory that could even culminate in the inception of ferroptosis (36). Moreover, the phototransduction cascade orchestrated by optic rod and cone cells hinges upon the meticulous governance of an ensemble of iron-laden proteins. Among these orchestrators is the heme-containing guanylate cyclase, a cardinal contributor to the harmonious progression of the phototransduction cascade (37). Iron’s pervasive influence extends, threading a continuum of intricate interactions crucial to retinal orchestration.

Iron homeostasis intricately intertwines with the complex tapestry of inflammation within the organism. The surfeit of Fe^2+^, whether via direct or indirect avenues, unfurls a cascade of events that propel the aberrant accumulation of reactive oxygen species (ROS) through the Fenton/Haber-Weiss reaction, thereby eliciting mitochondrial dysfunction. This tumultuous journey subsequently sets in motion the activation of IKK-β, a precursor to the stimulation of the NF-κB signaling pathway, thus fomenting the upregulation of key players such as tumor necrosis factor-alpha (TNF-α) and interleukin-6 (IL-6) (17, 29, 31–33, 38). Concomitantly, the trajectory navigated by protein kinase C-beta II (PKC-βII) embarks upon monitoring lipid peroxidation synthesis, orchestrating its activation in tandem with escalating intracellular ROS levels (39). It further steers the phosphorylation of Ser-36 on p66Shc, thus choregraphing the suppression of forkhead box protein O3A (FOXO3A), orchestrating a suppression of catalase (CAT) and manganese superoxide dismutase (MnSOD) expression (38). PKC-βII, in its multifarious role, amplifies the destructive implications that iron imparts upon cellular domains, concurrently activating long-chain acyl-CoA synthetase 4 (ACSL4) (39). The symphony of inflammation resonates further as TNF-α kindles the expression of bone morphogenetic protein 6 (BMP6) within the corridors of liver sinusoidal endothelial cells (LSECs) (40). Parallelly, IL-6 orchestrates the upregulation of hepcidin and ferritin expression (18). This intricate nexus, however, ushers forth repercussions that shatter the equilibrium of intracellular iron homeostasis, steered by the interplay of BMP6 and hepcidin within the realm of inflammatory factors.

Within the ambit of glucose metabolism, iron emerges as an indispensable cofactor and a pivotal carrier of electrons, priming the stage for a symphony of redox reactions and electron transfers (18). Pertinently, iron-sulfur clusters, heralded as “protein repair groups,” unfurl their intricate composition comprising iron and sulfur atoms. These elemental assemblages assume a role of paramount significance as the bedrock for the functional centers of an array of enzymes orchestrating the landscape of glucose metabolisms. Navigating the expanse of these enzymatic domains, we encounter luminaries such as NADH-ubiquinone oxidoreductase, succinate dehydrogenase, and cis-aconitase. This trio stands resolute within the intricate corridors of the tricarboxylic acid cycle, embellishing oxidative phosphorylation, and setting in motion the choreography of electron transfer along the mitochondrial respiratory chain (17). In this intricate network, iron unfurls its multifaceted contributions, culminating in the organization of an orchestra of metabolic transformations that underpin glucose metabolism.

Lipid metabolisms find themselves intricately woven into the tapestry of iron’s influence, unveiling an indelible nexus. In the realm of type 2 diabetes mellitus, research led by Chen et al. (41) has unveiled a discernible correlation between systemic iron levels and visceral fat mass. Sandro and colleagues (10), in their study involving Lepr^db/db^+Fpn^wt/C326S^ mice, have illuminated the occurrence of fatty acid deposition within the liver. Systemic iron has been noted to offer protective attributes against obesity in Hfe^-/-^ mice placed on a high-fat regimen, a counterintuitive observation augmented by the intriguing revelation that a high-iron diet might, in parallel, instigate elevated lipid synthesis (18). Not confined to direct participation in lipid oxidation, iron undertakes an intricate role in indirectly bolstering lipid metabolism through its sway over the expression of inflammatory factors, adiponectin, and leptin within adipocytes (9, 42, 43). This intricate interplay ultimately pivots upon the axis of inflammation, orchestrated by the diminution of adiponectin expression stemming primarily from the elevation in the expression of IL-6 and TNF-α (44). A cascading effect unfolds as this shift catalyzes the phosphorylation of cyclic-AMP response binding protein (CREB) and occupancy of the leptin gene promoter, culminating in the attenuation of leptin expression (42). This intricate interplay reverberates, fostering heightened appetite and curtailed lipolysis, thereby underpinning the association of a high-iron diet with escalated lipid synthesis. Curiously, the dynamic interplay of high iron levels may also impel an accelerated metabolic rate within tissues (45), thus inferring a relative fortification against obesity. In a seemingly contradictory twist, iron’s influence on overall body weight contrasts with its effect on local tissue and organ fat deposition. This paradox might stem from the repercussions of heightened iron levels, inducing augmented oxidative lipolysis within adipose tissue. This, in turn, leads to an influx of surplus fatty acids into the liver, thereby seeding fatty acid deposition within hepatic domains. The dichotomy between adequate and excessive iron supply, elucidating disparate outcomes, further deepens the complexity (27). Disentangling this intricate web, Harrison et al. (28) posit that while childhood obesity may bear an association with iron deficiency, such a link might be confined to extreme scenarios of iron insufficiency. Thus, the relationship between iron and lipid metabolism unveils itself as an intricate mosaic, governed by nuanced regulatory mechanisms. Simultaneously, the study led by Kusminski (46) accentuates the role of mitochondrial protein containing Asn–Glu–Glu–Thr (NEET) sequence, an iron-sulfur cluster-binding protein within the outer mitochondrial membrane. This participant orchestrates a suppression of iron transport to the mitochondrial matrix, thus retarding the pace of fatty acid β-oxidation and ultimately fostering the augmentation of adipose tissue. The influence of iron extends to the realm of adipocyte differentiation as well, with diminished iron levels within the adipocyte cytoplasm and mitochondria heralding the attenuation of adipose gene expression and lipid synthesis (34). In this intricate choreography, the resonance of adipose gene expression closely intertwines with the orchestra of iron-related genes, exemplified by cytosolic aconitase 1 (ACO1) (47). This cascade ushers forth a harmonious interplay within the realm of lipid metabolism and iron’s resounding influence.

Beyond its immediate role in nutrient oxidation, iron presides as a pivotal custodian, intricately overseeing the panorama of metabolic processes. It orchestrates the symphony of alterations in metabolic rate, the fine-tuning of fuel preferences, and insulin’s secretion and its ensuing actions (28). Within this discourse, we embark on a concise exploration of iron’s sway over insulin’s secretion and its intricate dance. The mediation of glucose-stimulated insulin secretion finds its nexus within the pathway governed by glutathione (GSH) through the conduit of the isocitrate-to-SUMO-specific peptidase 1 (48). Within this delicate choreography, two principal protagonists, GSH and isocitrate, emerge as pivotal players, both intimately entwined with iron’s influence. The surge of excess Fe^2+^ in the labile iron pool (LIP) triggers the production of a surfeit of hydroxyl radicals through the Fenton/Haber–Weiss reaction (26). This catalyzes a relentless ascent in intracellular oxidative stress. Under the microscope of Jingzhi et al. (20), this burgeoning oxidative stress reverberates as a diminishing melody for GSH production and the ratio of GSH to GSSG. The intricate tapestry unfolds, and the levels of intracellular GSH reveal a discernible inverse correlation with iron. Moreover, the synthesis of isocitrate weaves its narrative dependent upon cis-aconitase, a custodian of iron-sulfur clusters, its essential entity poised at the epicenter of activity. Notably, the synthesis of cis-aconitase pivots upon dynamic transformations (17, 49). As a concurrent participant within this tapestry, cis-aconitase is a member of the iron regulatory protein family, oftentimes referred to as IRP-1 (28). It operates within a nuanced realm: a deficit in intracellular iron prompts oxidative degradation of IRP-1’s iron-sulfur cluster, resulting in the eclipse of cis-aconitase activity (49). Thus, the cadence of its activity mirrors the oscillations within intracellular iron homeostasis, assuming its mantle as an important bridge between iron equilibrium and glucose metabolism. Iron’s reach extends to the intricate pathways of the insulin receptor signaling cascade, manifesting its influence through the promotion of IRS-1 phosphorylation and insulin phenylalanine residue hydroxylation. Akin to a resonating echo, the study by Altamura et al. (10) unveils a notable anomaly in Lepr^db/db^+Fpn^wt/C326S^ mice, marked by an exacerbated phosphorylation of IRS-1 compared to their Lepr^db/db^ counterparts, in parallel with a further reduction in AKT activation. This manifestation provides a poignant corroboration of the above delineation. Another facet unfurls as iron intertwines with the landscape of insulin resistance, wielding its power to hinder lipocalin expression by propelling FOXO1’s binding to the peroxisome proliferator-activated receptor gamma response element (PPRE). This intricate gambit exacerbates insulin resistance (34). Within this intricate theater, the PHD/HIF system unfurls its influence upon the process of glucose-stimulated insulin secretion within beta cells (50). While HIF-α famously wields its influence, augmenting glucose-stimulated insulin secretion by driving the expression of GLUT1 and GLUT3 (51), its influence dances on a precipice, also eliciting insulin resistance by disrupting AKT phosphorylation (52). A play of balance ensues as iron-induced oxidative stress fuels the proteasomal degradation of HIF-α, amplifying the activity of prolyl hydroxylase (PHD) (18). In culmination, whether entangled within excess or deficiency, the saga of intracellular iron intricately weaves its influence into the fabric of insulin secretion. Moreover, iron overload’s intricate tango with the PHD/HIF system unveils a multifaceted choreography. Thus, the mechanism underpinning insulin’s secretion and its subsequent actions echoes inextricably with the cadence of intracellular iron homeostasis.

## 3 Systemic and retinal iron homeostasis

**Figure 2:**
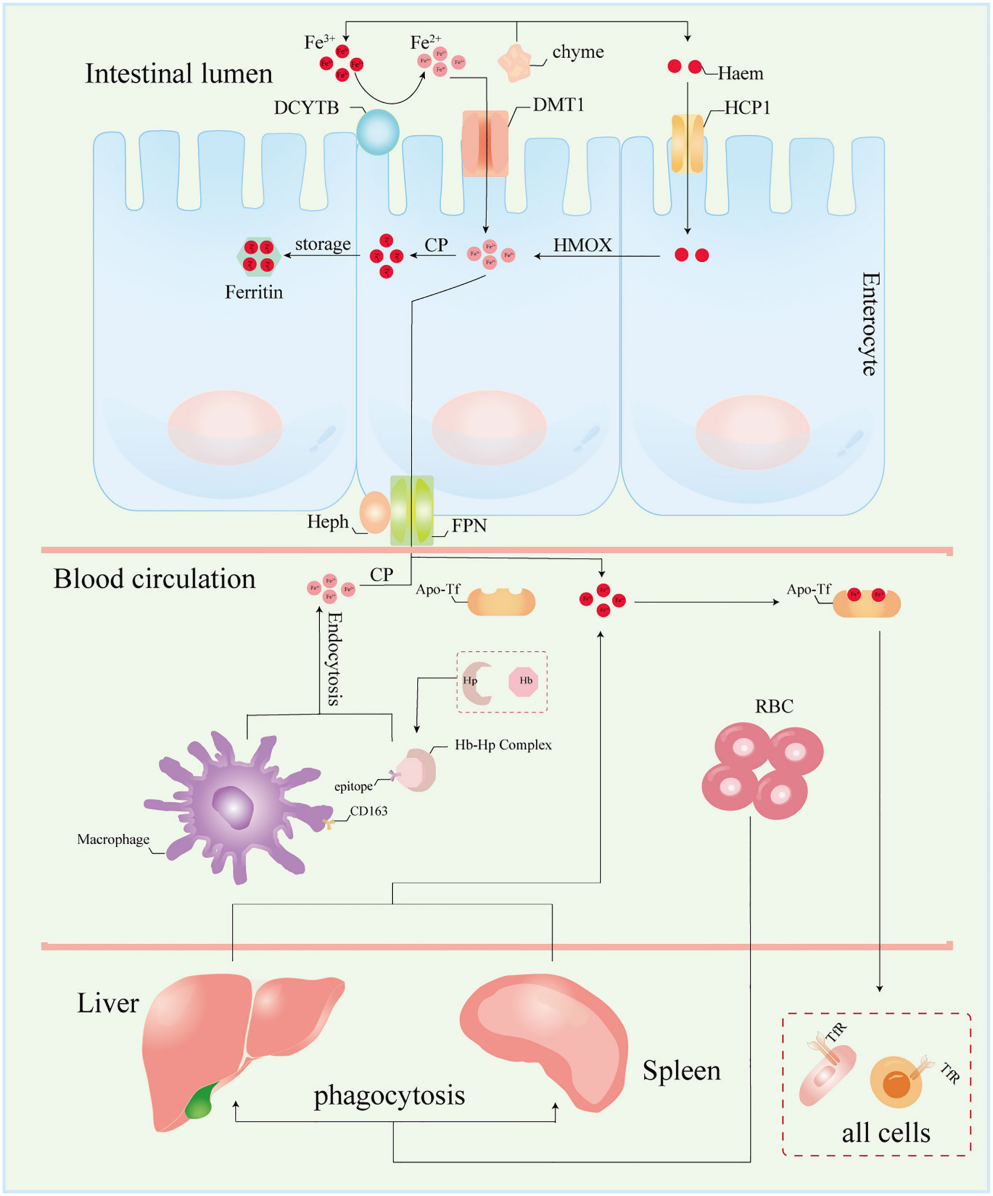
Patterns of iron absorption in intestinal cells. DCYTB, duodenal cytochrome b; DMT1, divalent metal ion transporter 1; HCP1, haem carrier protein 1; CP, ceruloplasmin; HMOX, heme oxygenase; Heph, hepcidin; FPN, ferroportin; Tf, transferrin; Hp, haptoglobin; Hb, hemoglobin; RBC, red blood cell; TfR, transferrin receptor.

A well-defined regulatory apparatus governing the exodus of iron across the human body remains elusive. The egress of iron largely hinges on a fraction allocated to the desquamation of expired cells, both in normal and pathological contexts, along with physiological and pathological blood loss, biliary excretion, and the renewal of intestinal epithelial cells. Notwithstanding, the lion’s share of iron is reclaimed by the tapestry of tissue cells. Hence, the orchestration of iron homeostasis pivots upon its acquisition, conveyance, and consumption within the organism. Within this tableau, two distinct routes beckon for cellular iron ingress: the non-transferrin-bound and the transferrin-bound conduits. In contrast, a solitary mechanism stands sentinel for cellular iron egress—the ferroportin-mediated pathway (**Figure 2**). Despite this orchestrated choreography, genes germane to iron metabolism unveil a tapestry of tissue-specific expression (**Table 1**). Yet, an uncharted realm rests unexplored: the intricate mediation by which tissue-specific expression unfurls its mantle of susceptibility to iron overload. Notably, the chorus of studies has underscored the nexus between tissue-specific architecture and vulnerability to the onslaught of iron overload-induced injury. This discourse, however, steers clear of delineating the interplay between tissue-specific gene expression and this predilection for vulnerability. Enshrined within the sanctuary of such specificity, the retina emerges as a poignant case study. The retina’s unique architectural framework renders it vulnerable to the machinations of hydroxyl radicals spawned by an excess of iron. Thus, the imperative to shepherd retinal iron supply within strict confines surfaces, to avert the structural upheaval unfurled by the sinister cloak of reactive oxygen species (ROS) accumulation (31). In this intricate dance, the blood-retina barrier (BRB) steps onto the stage as a necessary ally. Baumann et al., through the selective elimination of Müller cells in mice, dismantled the edifice of BRB, witnessing in its wake an affluent accrual of iron within the neural retina (55). This irrefutably underscores the indispensability of BRB in sculpting a meticulous command over retinal iron supply, masterminding an equilibrium within the expanse of retinal iron homeostasis.

**Table 1:** Tissue-specific expression of iron metabolism–related proteins (53, 54).

a												
	Tfr1 mRNA		Tfr2 mRNA		DMT1 mRNA			FtH mRNA	FtL mRNA		IRP1 mRNA	
principal organ	spleen		intestine and liver		intestine			kidney and heart	spleen and liver		kidney	
secondary organ	intestine		No		kidney and spleen			intestine, liver, and spleen	kidney		other tissues showed similar expression levels	
other	liver, heart, kidney, and lung		No		liver, lung, and heart			lung	heart and lung			
b												
	IRP2 mRNA		Hfe mRNA		Hepcidin mRNA			FPN1 mRNA	Hephaestin mRNA		ZIP14 mRNA	
principal organ	lung, liver, intestine, and heart		liver		liver			kidney, intestine, liver, and spleen	intestine		intestine	
secondary organ	spleen and kidney		other tissues showed similar expression levels		heart and intestine			heart and lung	lung and spleen		liver and heart
other					lung and kidney				heart, kidney, and liver		kidney	

Hfe, homeostatic iron regulator gene; IRP, iron regulatory protein; FPN, ferroportin; TfR, transferrin receptor; DMT1, divalent metal ion transporter 1.

**Figure 3:**
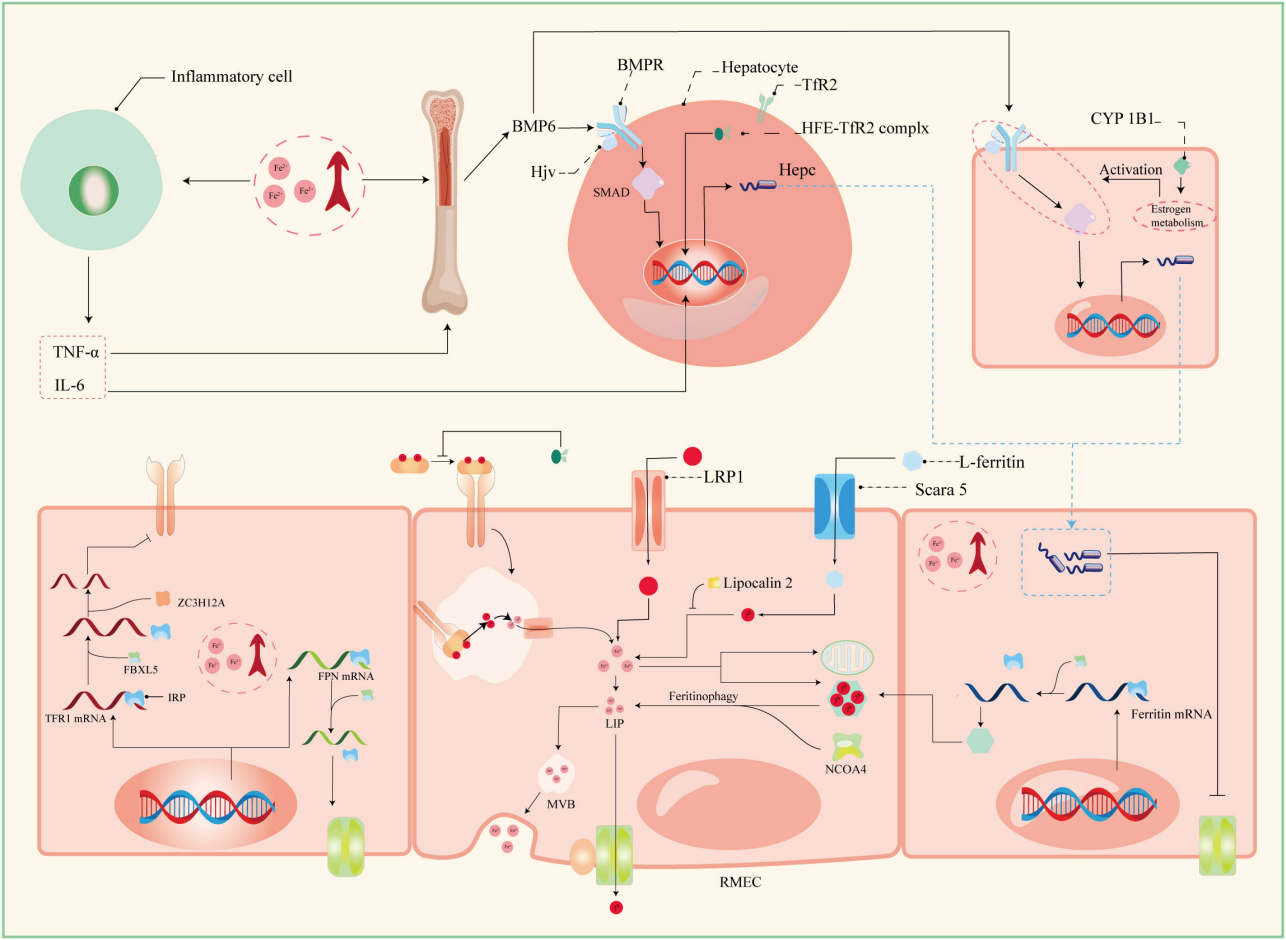
Patterns of iron absorption in RMEC. TNF-α, tumor necrosis factor α; IL-6, interleukin 6; BMP-6, bone morphogenetic protein 6; BMPR, bone morphogenetic protein receptor; Hjv, hemojuvelin; HFE, homeostatic iron regulator Gene; Hepc, hepcidin; CYP1B1, cytochrome P450 family 11 subfamily B member 1; ZC3H12A, zinc finger CCCH-type containing 12A; FBXL5, F-box and leucine-rich repeats protein 5; IRP, iron regulatory protein; IRE, iron responsive element; LRP1, low-density lipoprotein receptor-related protein 1; NCOA4, nuclear receptor coactivator 4; MVB, multivesicular body; Scara5, scavenger receptor class A member 5; RMEC, retinal microvascular endothelial cell.

Serum iron in humans primarily arises from two principal sources: the recycling of erythrocytes and intestinal absorption. However, intestinal uptake accounts for a mere 5–10% of the total, with the lion’s share originating from hemoglobin contained within aging erythrocytes (56). The absorptive duty falls upon duodenal and proximal jejunal enterocytes, predominantly facilitated by the divalent metal iron transporter-1 (DMT1), also known as SLC11A2, which graces the parietal membrane (17). This venture necessitates the participation of duodenal cytochrome B (DCYTB), situated on the parietal membrane of duodenal cells. DCYTB is instrumental in the reduction of Fe^3+^—a transformation vital for most dietary iron present in the form of Fe^3+^ (18). Of note, a fraction of iron ingested by enterocytes is directly obtained as heme from dietary sources, facilitated by the heme carrier protein 1 (HCP1) present in the enterocyte parietal domain (17). Upon their ingress into enterocytes, heme undergoes rapid iron release, a process reliant on the intervention of heme oxygenase (HMOX) (18). This sequence underscores the pivotal role of heme as a primary source of systemic iron. Senescent erythrocytes, on the other hand, undergo phagocytosis by macrophages resident in the liver and spleen, effecting the liberation of iron (56). Moreover, circulatory free hemoglobin forms HP-Hb complexes through binding with haptoglobin, heralding the exposure of new antigenic clusters. This culminates in the endocytosis of complexes by macrophages, fostering iron recycling, orchestrated by the scavenger receptor CD163 (57). In addition to this, free hemoglobin can traverse the threshold of the cytoplasm via the low-density lipoprotein receptor-related protein 1 (LRP1), heme-responsive gene protein 1 (HRG1), and FLVCR heme transporter 2. A notable facet of blood circulation is that Fe^3+^ constitutes the primary transported form of iron. This preference stems from the inherent stability of the half-filled 3d5 electron structure of Fe^3+^. This disposition finds apt companionship in the form of ferroportin (FPN) and hephaestin, both stationed on the cell membrane. These facilitators orchestrate the transfer of ferrous ions from enterocytes, hepatocytes, and macrophages into the systemic circulation (16, 17). However, due to its low water solubility, Fe^3+^ requires the aid of transferrin for its transportation. Transferrin serves as the conveyance vessel, transporting iron via the circulatory system to cells that express transferrin receptors, such as retinal endothelial cells (RECs) (16) (**Figure 3**). The fundamental mode of cellular iron uptake revolves around the classical transferrin-mediated pathway, emphasizing its pivotal role in governing intracellular iron homeostasis. In this orchestration, transferrin receptors (TFR) play a prominent part, with two main types distinguished: TFR1 and TFR2. TFR1, prominently stationed on the surface of most cell membranes, assumes the mantle of chief conduit for cellular iron uptake, while TFR2 is primarily distributed within hepatocytes (17). The entry of Fe^3+^-loaded transferrin into endosomes occurs through the agency of TfR1 and a clathrin-mediated endocytic process in retinal endothelial cells (ECs) (28). The endosomal milieu, with its pH below 5.6, its chelating agents, and the altered conformation of TfR1 on the membrane, collectively orchestrate the release of Fe^3+^ from transferrin. Subsequently, this Fe^3+^ is converted to Fe^2+^ with the participation of the six-transmembrane epithelial antigen of prostate 3 (STEAP3) (17). Following this transformation, Fe^2+^ embarks on a journey to the cytoplasm via the divalent metal iron transporter-1 (DMT1), also known as SLC11A2. Beyond the confines of the Tf-TFR1 pathway, recent inquiries have uncovered another avenue for the transport of free Fe^2+^. A minute fraction of free Fe^2+^ can navigate its way to retinal endothelial cells (ECs) through the zinc transporter 8 (ZIP8) and zinc transporter 14 (ZIP14) (16, 18). Likewise, employing similar mechanisms, retinal endothelial cells (RECs) and Müller cells shoulder the responsibility of iron distribution within the retina through the agency of ferroportin (FPN). Subsequently, various retinal cell types, including retinal pigment epithelial cells (RPECs), ganglion cells, bipolar cells, cone cells, rod cells, and additional bipolar cells, import iron via different pathways—TfR, DMT1, as well as Zip8 and Zip14. In addition to these classical pathways, L-ferritin assumes the role of an iron carrier into the retina, selectively binding to scavenger receptor class A member 5 (SCARA5) receptors on the surfaces of retinal endothelial cells (37). Simultaneously, H-ferritin can enter Müller cells, facilitated by TIM2 receptors (35). The meticulous control of retinal iron content is predominantly orchestrated by retinal pigment epithelium (RPE) and Müller cells. RPE, for instance, is equipped to engulf damaged or deceased photoreceptors (18), subsequently expelling iron to the choroid via the basal surface-associated FPN (17). Meanwhile, Müller cells facilitate the export of iron to the vitreous humor, again utilizing the FPN mechanism (17). Interestingly, in an unexpected twist, Ashok and colleagues (58) discovered that the prion protein (PrPC) also assumes the role of a ferrireductase partner, lending a helping hand in the transport of retinal iron through DMT1. Upon entry into cells, the iron is predominantly allocated to the synthesis of heme, iron-sulfur (Fe-S) clusters, and cytochrome c oxidase within the mitochondrial matrix. Some fraction of the remaining iron finds its place within ferritin, lying dormant in its inactive form. The rest constitutes the labile iron pool (LIP) in the guise of ferrous ions (13, 59). During times of necessity, ferritin releases iron to fulfill cellular iron requirements and maintain LIP levels, facilitated by the autophagy process. This intricate mechanism requires the involvement of nuclear receptor co-activator 4 (NCOA4), whose activity is augmented with the assistance of lysosomes (60). Notably, the delicate balance of LIP homeostasis bears relevance to the development of cellular ferroptosis. Should LIP become overloaded, the surplus Fe^2+^ is extracellularly expelled via the prominin2-multivesicular body (MVB)-exosome route, thereby safeguarding the integrity of intracellular iron equilibrium (59).

The establishment and maintenance of iron homeostasis within most cellular environments hinges upon the intricate interplay between iron regulatory proteins (IRPs) and iron-responsive elements (IREs), with their mutual binding and disassociation being modulated in response to shifts in intracellular iron concentrations (10). IREs constitute untranslated sequences embedded in mRNA, notably situated in key regions such as the 3’-UTR of transferrin receptor 1 (TFR1) mRNA, the 5’-UTR of ferritin mRNA, the 5’-UTR of ferroportin (FPN) mRNA, and the 5’-UTR of solute carrier family 11 member 2 (SLC11A2) mRNA. This configuration empowers IREs to exercise comprehensive command over the import and export of iron. Under conditions of iron excess, IRPs dissociate from IREs with the assistance of FBXL5 (33). This process results in distinct outcomes: when separated from the 3’-UTR of TFR1 mRNA, IRP reduces the structural stability of the mRNA, rendering it susceptible to cleavage by ZC3H12A and RC3H1 nucleases, thereby inhibiting translation. Conversely, the detachment of IRP from the IRE of FPN mRNA stimulates its translation and subsequently enhances iron export (33). Furthermore, the liver, an essential organ for iron storage in humans, plays a pivotal role in maintaining systemic iron homeostasis by producing substantial quantities of ferritin and hemosiderin. To orchestrate these tasks, a regulatory network encompassing the bone morphogenetic protein (BMP)/SMAD pathway, hemochromatosis protein (HFE), and hemojuvelin (HJV) comes into play (31). The iron-responsive BMP/SMAD signaling pathway effectively upregulates hepcidin expression in hepatocytes, with the involvement of HJV further modulating signal transduction accessibility and specificity by forming a complex with the BMP receptor (BMPR) as a co-receptor, with HJV being tethered to the cell membrane via glycosylphosphatidylinositol (GPI) linkage (10, 61). Notably, the expression of BMP-6, a key player in this pathway, is also observed in retinal pigment epithelial cells (RPECs) (62). The protein encoded by the HFE gene, a non-classical major histocompatibility complex class I (MHC I) protein, exerts its regulatory influence by competitively impeding the interaction between transferrin receptor 1 (TfR1) and transferrin. Moreover, it forms a complex with TfR2, jointly modulating the expression of hepcidin—a pivotal player in orchestrating intracellular iron equilibrium (61). The homogenous missense mutation C282Y within the HFE gene underpins hereditary hemochromatosis (HH), a condition that disrupts cellular iron homeostasis (61). Notably, the HFE protein predominately localizes to the basolateral membrane of retinal pigment epithelial cells (RPECs), alongside hepatocytes (63). In a noteworthy study, Chaudhary et al. (63) demonstrated that diabetic mice deficient in HFE exhibited a more pronounced disruption in blood-retinal barrier (BRB) integrity and greater neuronal cell loss compared to their diabetic counterparts without HFE deficiency.

Hepcidin, recognized as an antimicrobial peptide, consists of 25 amino acids, and operates as a peptide hormone. Its primary function involves constraining iron cycling and absorption through a negative regulatory mechanism. This is achieved by initiating endocytosis and lysosomal degradation of ferroportin (FPN) (18). Beyond its presence in hepatocytes, hepcidin is broadly expressed across various ocular cell types, including retinal pigment epithelial cells (RPECs), retinal endothelial cells (RECs), and Müller cells, among others (16). As a result, the Hepcidin-FPN axis significantly influences retinal iron homeostasis. Notably, an excess of systemic iron sensed by RECs triggers an upregulation of the BMP6/SMAD-Hepcidin-FPN axis. This, in turn, leads to a decrease in FPN abundance on the cell membrane, thereby disrupting retinal iron balance and fostering oxidative stress (32). Effective functioning of FPN relies on the presence of hephaestin (18). An insightful study by Zhang et al. (16) reported pathological iron accumulation in the neural retina of mRx-Cre^+^, Cp^-/-^, Heph^flox/flox mice^, relative to the control group. However, this accumulation exhibited a lesser degree of neuroretinal iron deposition when compared to systemic Cp^-/-^, Heph^-/-^ mice, and notably, did not manifest similar retinal degeneration. Additionally, the researchers observed concurrent changes in the expression of neuroretinal Zip8 and Zip14 in systemic Cp^-^, Heph^-/-^ mice, aligning with alterations in cellular iron levels. However, interestingly, iron accumulation was observed in the neural retina of mRx-Cre^+^, Fpn^flox/flox^ mice, as noted by (64). This observation prompted the consideration that hephaestin (heph) deficiency in the neural retina might contribute to elevated retinal Fe^2+^ levels in mRx-Cre^+^, Cp^-/-^, Heph^flox/flox^ mice. These increased Fe^2+^ levels could be attributed to the entry of Fe^2+^ into cells via the mechanisms mediated by Zip8 and Zip14, subsequently inducing aberrant iron deposition (16). It is conceivable that hephaestin’s role in the retina transcends its mere association with ferroportin (FPN) and extends to broader regulation of retinal iron homeostasis. Furthermore, PIEZO1, a mechanosensitive ion channel, emerges as a pivotal regulator influencing macrophage phagocytic activity, erythrocyte renewal, and hepcidin dynamics. In a study by Shang et al. (56), it was found that gain-of-function (GOF) expression of PIEZO1 in mice or macrophages had the potential to disrupt hepcidin levels. This disruption, in turn, led to iron overload in both mice and macrophages. Notably, lipocalin 2, a secreted glycoprotein, assumes a negative role in cellular iron homeostasis as it functions as a siderophore-binding protein. Lipocalin 2 can intricately bind to Fe^3+^, thereby impeding its conversion to Fe^2+^ with the assistance of catecholamines (28, 65).

CYP1B1, an integral member of the heme-containing monooxygenase family, possesses the capacity to catalyze monooxygenation reactions with the support of chaperone proteins, thereby imparting significance to retinal iron homeostasis. Beyond its presence in non-parenchymal liver cells, CYP1B1 is also structurally expressed in diverse components of the blood-retinal barrier (BRB), encompassing pericytes, endothelial cells (ECs), and astrocytes (66). In a notable proposition by Song et al. (17), CYP1B1 was suggested to wield the potential to modulate the activity of the BMP/SMAD signaling pathway by influencing estrogen metabolism within distinct BRB cellular constituents. This, in turn, could orchestrate the expression of hepcidin, thus orchestrating the governance of retinal iron homeostasis. Pertinently, the absence of CYP1B1 was shown to attenuate ischemic retinal neovascularization and restrain the expression of peroxisome proliferator-activated receptor γ (PPARγ). It has been documented in certain investigations (67, 68) that the deficiency or inhibition of CYP1B1 heightens retinal oxidative stress, concurrently diminishing the pro-angiogenic activity of ECs, and causing the decline of conventional enzymes linked to reactive oxygen species (ROS) generation. Nonetheless, the intricate molecular mechanisms that oversee the secretion of CYP1B1 within the retinal milieu, along with its interplay with retinal iron homeostasis, remain to be unequivocally elucidated.

## 4 Iron overload and DR

Initially, discrepancies in glucose metabolism were noted in individuals afflicted with hereditary hemochromatosis (HH), offering insight into the intricate connection between systemic pathological iron deposition and diabetes mellitus (DM) (69). A recent meta-analysis focusing on ferritin further underscores this correlation (70). Likewise, epidemiological surveys (71) and clinical investigations into HH and Thalassemia treatment echo a similar sentiment (28). However, it is worth noting that DM can also exert influence on systemic and retinal iron homeostasis. Altamura (10) et al. documented elevated serum iron levels in Lepr^db/db^ mice in comparison to wild-type counterparts, with even more pronounced elevation in Lepr^db/db^+Fpn^wt/C326S^ mice compared to Fpn^wt/C326S^ mice. Moreover, several studies (13, 20) have reported pathological iron accumulation within the retinas of diabetic mice. Zhang et al. (72) demonstrated that heightened glucose levels could trigger an increase in iron concentrations within human retinal endothelial cells (hRECs). Furthermore, some researchers employed Perls’ Prussian Blue staining for semi-quantitative analysis, revealing substantial abnormal iron accumulation in the retinal pigment epithelium (RPE) and outer plexiform layer (OPL) among patients with DR compared to healthy, age-matched controls (55). These collective findings underscore that the relationship between iron and diabetes is not a simplistic unidirectional association but rather a multifaceted bidirectional interplay. They also shed light on the potential involvement of iron in the onset and progression of DR, a topic that has garnered considerable critical attention.

It is widely recognized that the retina exhibits aberrant iron deposition in DR, likely attributable to retinal hepcidin upregulation prompted by systemic iron overload, subsequently leading to diminished expression of ferroportin (FPN) in the retina. Moreover, the diabetic milieu exposes cells to elevated glucose levels, fostering heightened inflammation, with inflammatory cytokines further enhancing hepcidin expression (73). Within the context of diabetes mellitus (DM), a prominent hallmark is mitochondrial dysfunction (74). Factors such as hyperglycemia, hypoxia, and inflammation collectively contribute to disrupted mitochondrial phagocytosis, culminating in the accumulation of dysfunctional mitochondria and perturbation of the mitochondrial network, thereby driving atypical mitochondrial dynamics (13). Given that the majority of intracellular iron enters mitochondria, the impaired functionality of these pathological mitochondria disrupts labile iron pool (LIP) homeostasis, thereby inciting pathological intracellular iron deposition. Notably, hyperglycemia itself leads to heightened heme catabolism, liberating significant quantities of free iron, while retinal hemorrhages further exacerbate iron dysregulation (75). The systemic iron overload resulting from DM prompts the upregulation of Zip8 and Zip14 expression in the neural retina. Consequently, an excess of Fe^2+^ infiltrates the retina through Zip8 and Zip14, thereby triggering retinal iron overload. Collectively, we posit that these factors outlined above may collectively contribute to the disruption of retinal iron metabolism observed in diabetic retinopathy.

The disruption of both the intracellular and extracellular microenvironments within the retina resulting from retinal iron overload and systemic iron overload causes significant harm to the equilibrium of the stress/anti-stress system. The presence of abundant retinal polyunsaturated fatty acids (PUFAs) combined with its distinct photo-oxidative milieu renders the retina highly vulnerable to the detrimental effects of hydroxyl radicals generated due to iron imbalance. This heightened susceptibility contributes to the manifestation of structural and functional pathological alterations.

### 4.1 Iron overload, MD, and ER stress in the retina

Mitochondrial dysfunction (MD) stands as a prominent hallmark of diabetes (74), playing a pivotal role in triggering oxidative stress in endothelial cells (ECs) (26). Moreover, MD disrupts intracellular iron homeostasis, while concomitant iron overload intensifies oxidative stress (OS) and the peroxidation of membrane lipids. This, in turn, interferes with ATP synthesis and exacerbates the permeability of both inner and outer mitochondrial membranes (60). This intricate interplay engenders a detrimental cycle, culminating in elevated oxidative stress and an onset of inflammatory factors within the retina (60, 76). Iron is intrinsically intertwined with retinal oxidative stress and heightened inflammation levels due to the mediation of MD.

The endoplasmic reticulum (ER) is widely recognized as a pivotal hub for intracellular protein folding and maturation. ER stress induces protein misfolding, thereby triggering the unfolded protein response (UPR) to reinstate cellular homeostasis (77). However, persistent ER stress that exceeds a certain threshold leads to UPR failure, instigating an influx of pro-inflammatory factors and provoking cell death responses (78). ER stress has been intimately associated with the progression of DR, wherein high glucose (HG) induces ER stress through mitochondrial dysfunction and the activation of NADPH oxidase (79). Recent findings by Wang et al. (80) have demonstrated that HG can elevate ER stress by upregulating MALAT1 expression, thereby increasing the levels of TNF-α and IL-6. Simultaneously, ER stress can trigger NCOA4-mediated ferritin autophagy, culminating in iron overload, while concomitantly suppressing PPARγ expression, thereby enhancing the susceptibility to ferroptosis (77, 81). Furthermore, ER stress contributes to the activation of the BMP/SMAD signaling pathway by inhibiting TMPRSS6 and stabilizing hepcidin mRNA through the RNA-binding protein HuR. This ultimately stimulates hepcidin expression, thereby disrupting intracellular iron metabolic homeostasis (82). More recently, Ning et al. have identified ER stress as an upstream signal for ferroptosis (83). Hence, the etiological underpinnings of retinal ferroptosis in the context of diabetic retinopathy have been further elucidated.

The retina, an integral component of the central nervous system, possesses a constrained ability for self-renewal and regeneration. The advancement of DR is significantly compounded by both MD and ER stress, which synergistically contribute to iron overload, oxidative stress, energy insufficiency, an inflammatory surge, and aberrant unfolded protein response (UPR).

### 4.2 Iron overload and retinal inflammation

Previous investigations have unveiled that an intracellular surplus of Fe^2+^ triggers oxidative stress through the Fenton reaction, subsequently stimulating IKKβ and thereby activating the NF-κB signaling pathway. The inhibitory κB (IκB) kinase (IKK) complex comprises two serine/threonine protein kinases, namely, IKKα and IKKβ, alongside a regulatory subunit known as NEMO (IKKγ), which aptly senses and integrates diverse stimuli. Notably, within this complex, IKKβ stands out as a key player in initiating the nuclear factor kappa-B (NF-κB) signaling pathway through its phosphorylation of IκBα (12). Recent findings have added another layer of complexity to IKKβ’s role. Specifically, it has come to light that the kinase domain of IKKβ can also phosphorylate AMPK, thereby eliciting potent anti-inflammatory effects. Remarkably, this AMPK phosphorylation exerts its anti-inflammatory influence without hindering IKKβ’s own function, thus offering a potential avenue for mitigating inflammatory damage provoked by lipopolysaccharides (LPS) (84). Thus, the equilibrium of inflammation levels is intricately regulated by IKKβ through its dual actions of phosphorylating IκBα and AMPK. However, a perplexing question arises: given that IKKβ has two distinct downstream pathways—one anti-inflammatory and the other pro-inflammatory—how does the sole stimulation of IKKβ ensure the activation of the pro-inflammatory pathway? An elucidation of this phenomenon has been provided by Zhang and colleagues (85). Their research highlights that high glucose (HG) conditions induce the concentration-dependent overexpression of TRIM46 in human retinal capillary endothelial cells (HRCECs). Importantly, TRIM46 amplifies the ubiquitination of IκBα, leading to protease-dependent degradation of IκBα, thereby liberating NF-κB. Based on these findings, we put forth a hypothesis that the hyperglycemic environment induces excessive iron accumulation in the retina, thus stimulating IKKβ in human retinal endothelial cells (hRECs). Furthermore, TRIM46 appears to tilt the balance in favor of pro-inflammatory signaling, thereby fostering a pro-inflammatory milieu.

In the context of DR, the concurrent presence of hyperglycemia and iron overload disrupts the process of mitochondrial phagocytosis within retinal cells and elevates the permeability of mitochondrial membranes. Consequently, damaged mitochondria release mitochondrial DNA (mtDNA) into the cytoplasm (13). Notably, oxidized mtDNA, serving as damaged associated molecular patterns (DAMPs), becomes susceptible to recognition by cytosolic pattern recognition receptors (PRRs) such as Toll-like receptor 4 (TLR4), Toll-like receptor 9 (TLR9), and NOD-like receptor family pyrin domain containing 3 (NLRP3) inflammasomes. This interaction prompts the activation of pro-IL-1β and pro-caspase-1, key elements of the pro-inflammatory response (76). Interestingly, the retinal vasculature in mice displays significant expression of the stimulator of interferon genes (STING) protein (86). Moreover, mtDNA has the capacity to activate cyclic GMP-AMP synthase (cGAS), which augments the signaling of STING and subsequently triggers the release of interferons. This cascade leads to the escalation of interferon levels in the retinal environment (60).

Irrespective of the alterations in retinal inflammatory signaling pathways observed in DR, HG consistently emerges as the primary instigator. Furthermore, iron significantly contributes as a prominent accomplice in exacerbating the pathological processes.

### 4.3 Iron overload and BRB structural disorder

The human retina, an integral component of the central nervous system, is characterized by an intricate network of nerves and blood vessels. Owing to the distinctive features of its internal architecture and external milieu, substances traversing into and out of the retina undergo stringent scrutiny by the blood-retinal barrier (BRB). Thus, preserving the structural integrity of the BRB is of paramount importance for upholding metabolic equilibrium, physiological composition, and functional integrity within the retina. Comprising pertinent cells and connexin components (8), the BRB’s structural perturbations are a significant contributor to the initiation and progression of DR. The interplay between iron overload and the BRB is far from being a mere unidirectional connection. Both systemic and retinal iron excess can disrupt the structural integrity of the BRB, transforming it into a “porous” state that facilitates unregulated iron entry from the systemic circulation into the retina. As such, delving into the intricate relationship between iron and BRB disruption holds promise for identifying novel therapeutic targets in the quest to combat DR.

The cellular constituents of the blood-retinal barrier (BRB) primarily encompass retinal pigment epithelial cells (RPECs), retinal endothelial cells (RECs), pericytes, and Müller cells. RPECs, integral to the outer blood-retinal barrier (oBRB), are chiefly responsible for furnishing photoreceptors with oxygen and metabolic substrates, while also participating in the recycling of retinol through the phagocytosis of impaired photoreceptor outer segments (76). RECs and pericytes, on the other hand, constitute the principal cellular components of the retinal microvasculature (87). RECs orchestrate the selective regulation of plasma substances and cellular ingress into the retina, alongside the secretion of diverse cytokines that contribute to microvascular homeostasis. Concurrently, pericytes undertake secretory and contractile functions, providing structural support to endothelial cells (ECs), governing their proliferation and migration, and overseeing the regulation of retinal microvascular blood flow (9). Müller cells, as the prominent glial cells of the retina, extend their reach across the retinal landscape, serving the crucial roles of furnishing neuronal nourishment and disposing of metabolic waste to uphold the microenvironmental equilibrium (55). Notably, these cells are key contributors to the distribution of iron within the retina, facilitated by the production of hepcidin. Moreover, they engage in the secretion of “gliotransmitters,” which act as regulatory agents in modulating neuronal function (55). Intercellular tight junction (TJ) proteins, encompassing connexin components, stand as pivotal molecular entities that underpin the integral structural integrity of the blood-retinal barrier (BRB). Among these, zonula occludens-1 (ZO-1), claudin-5, and occludin emerge as notable constituents. The harmonious equilibrium within these cellular constituents, coupled with the robust presence of TJ proteins, collectively dictate the efficiency and efficacy of BRB functions. Pertinently, the imposition of iron overload precipitates structural perturbations within the BRB. This perturbation is orchestrated through the disruption of cellular architecture and the attenuation of TJ protein expression, orchestrated via diverse signaling pathways (75). In the ensuing discourse, we will expound upon the intricate mechanisms through which the pathological accumulation of reactive iron within the retina culminates in the disarray of endothelial cells (ECs) and the associated TJ proteins.

Across each constituent cellular element forming the intricate tapestry of the blood-retinal barrier (BRB), the foremost repercussion of iron overload is the induction of ferroptosis, precipitating an insidious cascade of structural disarray within the BRB. The elucidation of the intricate mechanisms underlying the occurrence of ferroptosis shall be expounded upon in the subsequent segment. Within the context of type 2 diabetes mellitus (T2DM), a pivotal hallmark is the emergence of insulin resistance (IR), wherein aberrations in the signaling pathways downstream of the insulin receptor stand as prime contributors to this pathophysiological state. Recent years have witnessed a growing body of research illuminating the propensity of DM to bestow systemic or cellular iron overload. Within this milieu, escalated intracellular Fe^2+^ concentrations engender a surge in the generation of reactive oxygen species (ROS), thereby provoking the phosphorylation of serine residues in insulin receptor substrates-1 (IRS-1) and disrupting the downstream signaling cascades orchestrated by IRS-1 (10, 18). In tandem, hydroxyl radicals exert their influence by hydroxylating insulin phenylalanine residues, thereby perturbing the efficacious engagement of insulin receptors (10). Paramount in its significance, iron overload emerges as a catalyst for mitochondrial dysfunction, orchestrated through the disruption of mitochondrial autophagy (88). Pathological perturbations within the mitochondrial milieu impart a notable elevation in the abundance of free fatty acids (FFAs), a phenomenon with repercussions of consequence. FFAs wield the capacity to enhance the phosphorylation of serine residues within insulin receptor substrates-1 (IRS-1), thereby orchestrating a disruption within the cascades of downstream signaling mediated by IRS-1 (22, 23). Intriguingly, recent investigations have unveiled an intriguing facet: FFAR1’s participation in the insulin secretory response subsequent to acute FFA treatment in murine subjects. However, the semblance of this effect eludes detection following chronic FFA exposure (89). Emerging as a pivotal nexus, endothelial nitric oxide synthase (eNOS) activation surfaces as an entity under the purview of insulin’s control, an influence intertwined with the functional vigor of endothelial cells (ECs). Moreover, oxidative stress (OS) perpetrates an untethering of eNOS, engendering a decline in nitric oxide (NO) synthesis (90). Within this construct, the conjecture emerges that iron overload, by virtue of its presence, might exacerbate the impairment of NO-mediated functionality within retinal endothelial cells (RECs), compounded by the confluence of insulin resistance and eNOS uncoupling, thus culminating in the structural disruption of the blood-retinal barrier (BRB).

The augmented permeability of retinal endothelial cells (RECs) engenders a consequential fluid exudation from the plasma into the retina, precipitating macular edema—a cardinal hallmark in the trajectory of DR. This phenomenon is underpinned by the disruption of intercellular tight junctions (85). Concurrently, the surge in expression of vascular endothelial growth factor (VEGF) and tripartite motif 46 (TRIM46) assumes a pivotal role in the structural dismantling of RECs, ushering the escape of retinal microvessel contents. Perturbed metabolic dynamics in the context of hyperglycemia orchestrate a surge in succinate levels, a state exacerbated by iron overload that augments the expression of GPR91. The latter, operating as a succinate receptor within the ambit of G protein-coupled receptors, is noteworthy in this interplay (75). Succinate, serving as a physiological agonist for GPR91, is thus the fulcrum upon which this interaction pivots. In the cascade that follows, these alterations collaboratively activate extracellular signal-regulated kinase 1/2 (ERK1/2), p38 mitogen-activated protein kinase (MAPK), and c-Jun N-terminal kinase (JNK) signaling, constituting a nexus framed within the succinate/GPR91 axis. These events precipitate the release of VEGF from retinal ganglion cells, solidifying their role in the intricate orchestration of DR pathology (75). In addition, the oxidative stress (OS) incited by iron overload exerts an additional impetus on the overexpression of vascular endothelial growth factor (VEGF) (35). This malignant upregulation of VEGF accentuates the degradation of tight junction (TJ) proteins, including zonula occludens-1 (ZO-1), claudin-5, and occludin, along with augmenting the abundance of caveolae on the membranes of retinal endothelial cells (ECs). This exacerbates the breach in the blood-retinal barrier (BRB) and consequent retinal edema (35). Concurrently, the influence of tripartite motif 46 (TRIM46) extends to RECs, where it catalyzes membrane lipid peroxidation and an inflammatory cytokine cascade. This is primarily achieved by orchestrating the ubiquitination and subsequent degradation of glutathione peroxidase 4 (GPX4) and inhibitory kappa B alpha (IκBα), thereby reducing cellular transmembrane electrical resistance (TEER) and elevating permeability (12). Moreover, TRIM46 overexpression engenders a decline in the levels of ZO-1 and occludin, mediated through the nuclear factor-kappa B (NF-κB) signaling pathway (12). While endothelial cells (ECs) bear pivotal significance in the retinal microvasculature, their role within the context of DR extends beyond a solitary role. Indeed, the dynamic interplay between pericytes and ECs governs the formation, maturation, and stability of the retinal microvasculature. Pericytes assert their influence on ECs by modulating the barrier functionality and upholding BRB stability through direct cell-to-cell contact or paracrine signaling. This orchestrated communication is facilitated by signaling pathways such as transforming growth factor-beta (TGF-β)/GFRB, angiopoietin 1 (Angpt1)/Tie2, and vascular endothelial growth factor A (VEGF-A)/VEGFR2 (87). Conversely, the repercussions of iron overload manifest as oxidative stress and inflammatory cascades, culminating in pericyte demise and the disruption of pericyte-endothelial cell (EC) signaling. This intricate perturbation yields the structural dismantling of the blood-retinal barrier (BRB) (9). Similarly vital to retinal equilibrium, retinal pigment epithelial cells (RPECs) encounter oxidative impairment due to iron overload, accelerating the decline of circular RNA SPECC1 (circSPECC1). This accelerates the manifestation of ultrastructural anomalies, cellular hypertrophy, and potential atrophy (91). These diverse morphological deviations collectively contribute to structural impairment of the outer blood-retinal barrier (oBRB). Furthermore, the activation and recruitment of microglial cells, coupled with the diminished expression of Rpe65 protein, conspire to render retinal pigment epithelium (RPE) cells functionally incapacitated or even deceased (91). The integrity of tight junctions (TJ) is pivotal for upholding RPE polarization, a factor essential for effective RPE junction maintenance and regulation of phagocytic activity. Notably, the deficiency of circSPECC1 significantly diminishes the formation of zonula occludens-1 (ZO-1) on the RPE cell membranes, thereby instigating depolarization of RPEs (91).

## 5 Ferroptosis and DR

**Figure 4:**
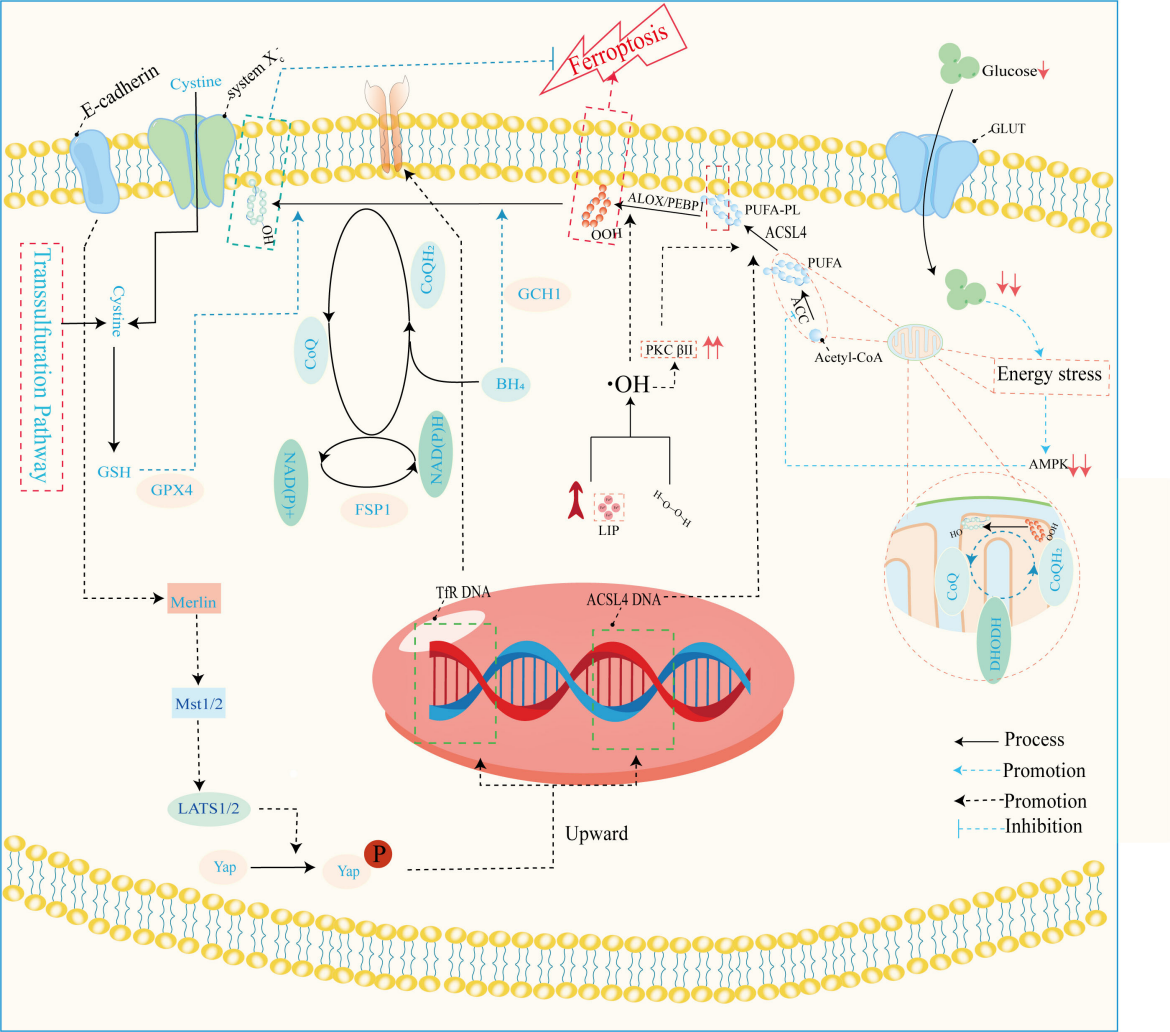
Mechanisms of ferroptosis regulation. This image mainly refers to Stockwell’s (59), Zhu‘s (93), and Wu’s (94) studies. GLUT, glucose transporter; ALOX, arachidonate lipoxygenase; PEBP1, phosphatidylethanolamine binding protein 1; ACLS4, acyl-CoA synthetase long-chain family member 4; PUFA, polyunsaturated fatty acid; PUFA-PL-OOH, phospholipid with peroxidized polyunsaturated fatty acyl tail; ACC, acetyl-coenzyme A carboxylase; AMPK, adenosine-monophosphate-activated protein kinase; DHODH, dihydroorotate dehydrogenase; CoQ, coenzyme Q; CoA, coenzyme A; PKCβII, protein kinase C beta type isoform 2; LIP, labile iron pool; BH_4_, tetrahydrobiopterin; NADPH, reduced nicotinamide adenine dinucleotide phosphate; NADH, reduced nicotinamide adenine dinucleotide; FSP1, ferroptosis suppressor protein 1; GPX4, glutathione peroxidase 4; GSH, glutathione; Mst1/2, macrophage stimulating 1/2; LATS1/2, large tumor suppressor1/2; Yap, yes1 associated transcriptional regulator; system X_c_^-^, anionic amino acid transport system.

Ferroptosis, a dire consequence of intracellular iron overload, represents a novel variant of regulated cell death (RCD) distinct from apoptosis, and it operates independently of caspases and the gasdermin (GSDMD) family. Its cardinal trait revolves around the pathological accumulation of intracellular iron and lipid peroxides, serving as drivers of orchestrated cell demise (59, 92) (**Figure 4**). Of paramount significance is the profound ultrastructural degradation of mitochondria, setting it apart from other forms of cell death. This distinctive signature entails mitochondrial atrophy, a reduction in mitochondrial cristae density, and the perturbation of membrane potential (19, 95). The nexus between ferroptosis and mitochondrial dysfunction (MD) assumes a complexion characterized by complexity, multifaceted interactions, and interwoven relationships. This orchestrated cell death process is contingent upon the concentration of intracellular iron, whereby surplus Fe^2+^ potentiates escalated oxidative stress and lipid peroxidation, thereby hastening the advent of ferroptosis. Moreover, the compounding factors of hyperglycemia and heightened levels of interleukin-1β (IL-1β) have been reported to activate p53, culminating in the inhibition of SLC7A11 expression. This suppression, in turn, impairs the synthesis of system Xc^-^ and precipitates the initiation of ferroptotic processes within cells (96). In a recent investigation, it was observed that high glucose (HG) exposure instigated a reduction in the mRNA levels of genes associated with proliferation (MELK, PLK1, PLK4, and CCNB2), accompanied by an elevation in ferroptosis-associated biomarkers (membrane lipid peroxides, mitochondrial superoxide, total and ferrous iron), as well as oxidized glutathione (GSSG) levels within ARPE-19 cells. Notably, the application of ferrostatin-1 exhibited a capacity to mitigate cellular demise in this context (5, 97). Hence, both metabolic alterations and inflammatory responses contribute as integral components. In the annals of past research, it is evident that apoptosis alone does not account for the entirety of cell death processes within the retina. Instead, pyroptosis, necroptosis, NETosis, and autophagy each contribute to varying degrees (20, 92). A comprehensive overview of the distinctions among these modalities is concisely presented in **Table 2**. It is worth noting that oxidative stress and inflammatory cascades stand as common denominators underlying these various forms of cell death (29). Iron overload has the capacity to generate excessive reactive oxygen species (ROS) via the Fenton reaction. Consequently, iron is intrinsically interwoven with various forms of cell death, playing a pivotal role in the initiation of such processes, as illustrated in **Table 2**. However, while iron occupies a significant place within the realm of various cell death mechanisms, it takes on a distinctive prominence specifically in the context of ferroptosis. Notably, iron’s influence is not only limited to being a primary instigator; it also serves as a triggering element for a spectrum of other cell death modalities. Although iron’s connection with other cell death pathways has been elucidated, its role, while vital, may not be the singular determinant, especially when considering cell death processes in the broader context. Notably, in the intricate landscape of DR, the interactions between iron and these cell death pathways are not yet fully characterized. However, evidence suggests that such connections might indeed exist, as supported in the context of retinal detachment, which shares similarities with DR in terms of disease progression. As retinal detachment can occur in advanced stages of both DR and other retinal pathologies, we posit that these associations might extend to the early stages of DR as well. Further exploration is warranted to comprehensively unravel these intricate interplays and their potential implications in the onset and progression of DR. The retina, a tissue rich in photoreceptors and characterized by its high content of polyunsaturated fatty acids (PUFAs), is particularly vulnerable to oxidative stress. Light-induced generation of reactive oxygen species (ROS) in photoreceptors, coupled with the influx of ferrous ions, underscores the retina’s susceptibility to iron-mediated oxidative damage. Iron’s participation is further pronounced due to its pivotal role in phototransduction within the retina. Given these attributes, the retina is highly sensitive to the perturbing effects of iron-induced oxidative stress. Notably, both clinical observations in DR patients and findings from animal studies underscore the potential for diabetes to trigger iron accumulation within the retina. This abnormal iron buildup disrupts the delicate equilibrium, setting the stage for pathophysiological changes. Strikingly, a shared attribute between DR and the phenomenon of ferroptosis is their reliance on ROS accumulation, fostering cellular oxidative stress. Concurrently, the retina’s abundance of PUFA—a substrate for ferroptosis—adds an intriguing dimension. Consequently, the hypothesis gains ground, in that ferroptosis may wield a more substantial influence in the progression of DR, particularly in the presence of iron overload. Further investigations are warranted to illuminate the intricate interplay between iron, oxidative stress, PUFA metabolism, and ferroptosis in the context of DR’s development.

**Table 2:** The differences of cell death. The cell death is associated with iron (92, 98).

	apoptosis	pyroptosis	necroptosis	ferroptosis
Definition	Programmed cell death depending on MOMP or caspase-8 activation, and subsequent caspase-3 activation.	Regulated necrosis depending on the caspase activation and the formation of plasma membrane pores by gasdamin families.	Regulated necrosis depending on MLKL and RIPK3, and in some contexts, RIPK1.	Regulated necrosis depending on lipid peroxides required for PUFAs, redox-active iron, and/or GPX4 dysfunction.
The roles of iron	ROS can oxidize cardiolipin and induce mitochondrial outer membrane permeabilization (MOMP) and cytochrome c release. Then, caspase 9 (CASP9) and caspase 3 (CASP3) are activated and induce apoptosis. Meanwhile, iron overload inhibits the formation of the short form of fas, which inhibits caspase 8 (CASP8)-dependent apoptosis. In addition, ROS can activate the apoptosis signal-regulating kinase 1 (ASK1)-p38/c-Jun N-terminal kinase (JNK) pathway to induce apoptosis.	ROS causes oxidation and oligomerization of the mitochondrial outer membrane protein Tom20. Then, bax is recruited to mitochondria by Tom20 and induces cytochrome c release and caspase 3 activation, which triggers the cleavage of gasdermin D (GSDME) and induces pyroptosis.	Iron overload induces overexpression of inflammatory factors such as TNFα, which bind to TNFR1 and form a signaling complex with the TNFR1-associated death domain (TRADD), RIPK1, and the small GTPase Rac1, thereby activating RIPK1/3 and JNK. TNFR1 coupled to riboflavin kinase (RFK) mediates the binding of FAD to NADPH oxidase 1, which activates NOX1, which activates the receptor-interacting serine protein, receptor-interacting serine-threonine kinase 1/3 (RIPK1/3), and mixed-lineage kinase domain-like (MLKL) to induce necroptosis.	Iron-dependent lipid ROS

The disruption of iron metabolism constitutes a highly destabilizing factor for retinal cells and serves as a pivotal catalyst for the initiation of ferroptosis. Among the key orchestrators of ferroptosis, acyl-CoA synthase long-chain family member 4 (ACSL4) and lysophosphatidylcholine acyltransferase 3 (LPCAT3) assume prominent roles (72). Additionally, the E-cadherin/merlin/Hippo/Yap pathway emerges as a critical regulator governing their expression (94). Of particular significance, ACSL4’s activation spurs the PUFA/membrane phospholipid axis into action, priming the cellular environment with substrates for lipid peroxidation. Notably, the synthesis of PUFA within this process hinges upon the concerted action of acetyl coenzyme A carboxylase (ACC). Notably, the cell’s energy stress sensing receptor, AMP-activated protein kinase (AMPK), exerts its control over ACC levels, thus contributing to the regulation of this intricate process.

Lipid peroxidation, a critical phenomenon, comprises two distinct pathways—enzymatic and non-enzymatic reactions. The enzymatic pathway, prominently executed by the lipoxygenase (LOX) family, initiates the exposure of carbon center radicals in polyunsaturated fatty acid phospholipids (PUFA-PL). Subsequently, these radicals react with hydroxyl radicals, leading to the formation of perilous polyunsaturated fatty acid phospholipid hydroperoxides (PUFA-PL-OOH) (99, 100). This reaction escalates the permeability and disrupts the integrity of cellular and mitochondrial membranes, pivotal for the onset of ferroptosis. The ferroptosis resistance system is a complex network of pathways, including the system Xc^-^/GSH/GPX4, FSP1/CoQ/NAD(P)H, GCH1/BH4/BH2, DHODH/CoQ, and transsulfuration pathways (59, 93). Among these, the most pivotal constituents are the glutathione (GSH)/glutathione peroxidase 4 (GPX4) and coenzyme Q (CoQ)/CoQH2 systems. These systems collectively orchestrate cellular defenses against the cascading events of ferroptosis, thus playing a significant role in maintaining cellular integrity and function. Resistance mechanisms play a pivotal role in counteracting the detrimental effects of lipid peroxidation, particularly in ferroptosis, where they attenuate the toxicity by reducing polyunsaturated fatty acid phospholipid hydroperoxides (PUFA-PL-OOH) to their non-toxic counterparts, PUFA-PL-OH (101). This function is tantamount to suppressing the “trigger button” of ferroptosis. Dysfunctional resistance systems, however, propel the process toward ferroptosis induction. In the context of pathological metabolism and inflammation resulting from diabetes mellitus (DM), intracellular iron homeostasis is disrupted. This disruption is accompanied by the concurrent impairment of the resistance systems. This dual effect exposes retinal cells to a precarious state. Among these cells, microvascular endothelial cells (ECs) are particularly susceptible to hyperglycemic conditions (72). In the progression of DR, the demise of microvascular ECs assumes a pivotal role as an initiator. Given that ECs form a fundamental cellular component of the blood-retinal barrier (BRB) (8), their demise leads to the structural upheaval of it. Consequently, the retina is deprived of its protective barrier system, and unrestricted access of serum iron to the retina becomes feasible. Notably, the death of ECs is discernible in non-proliferative diabetic retinopathy (NPDR), providing tangible evidence for the aforementioned concept (30). This underscores the critical involvement of ECs and their demise in the intricate cascade of events that characterize the early stages of diabetic retinopathy. Efficient functioning of photoreceptor cells, integral to visual perception, relies heavily on a steady supply of oxygen and nutrients from choroidal capillaries. This crucial task is undertaken by endothelial cells (ECs) and the retinal pigment epithelium (RPE), responsible for the delivery of these essential fuels to photoreceptors. The impairment of ECs and dysfunction of the RPE disrupt this delicate supply chain, leading to photoreceptor cells enduring a state of “starvation” and eventual demise. Intriguingly, iron, a pivotal player in cellular processes, is predominantly distributed within the RPE and photoreceptors (31). This allocation renders them particularly sensitive to the initiation of ferroptosis. Although the precise mechanisms orchestrating the development of ferroptosis in the context of DR remain incompletely elucidated, advancements have been achieved in understanding the molecular underpinnings governing the upregulation of acyl-CoA synthase long-chain family member 4 (ACSL4) and the disruption of diverse antioxidant systems. These insights pave the way for unraveling the intricate interplay between photoreceptor health, iron distribution, and the pathological processes inherent to DR.

In summary, we provide a comprehensive exploration of the intricate molecular biological mechanisms through which high glucose (HG) intricately governs ferroptosis in distinct retinal cell types, including retinal endothelial cells (RECs), retinal pigment epithelial cells (RPECs), and photoreceptor cells. Our investigation places special emphasis on elucidating the pivotal contributions of various small molecules, shedding light on the involvement of mitochondrial dysfunction (MD) both in instigating ferroptosis and deactivating the ferroptosis resistance system. Through a detailed examination of these mechanisms, we aim to unravel the complex interplay between HG and ferroptosis regulation, offering insights into the multifaceted responses of retinal cells to HG-induced stress. This knowledge promises to expand our comprehension of the intricate molecular pathways implicated in retinal pathophysiology, while fostering opportunities for targeted interventions to mitigate the adverse effects of HG on retinal health.

### 5.1 Non-coding RNA and ferroptosis

**Figure 5:**
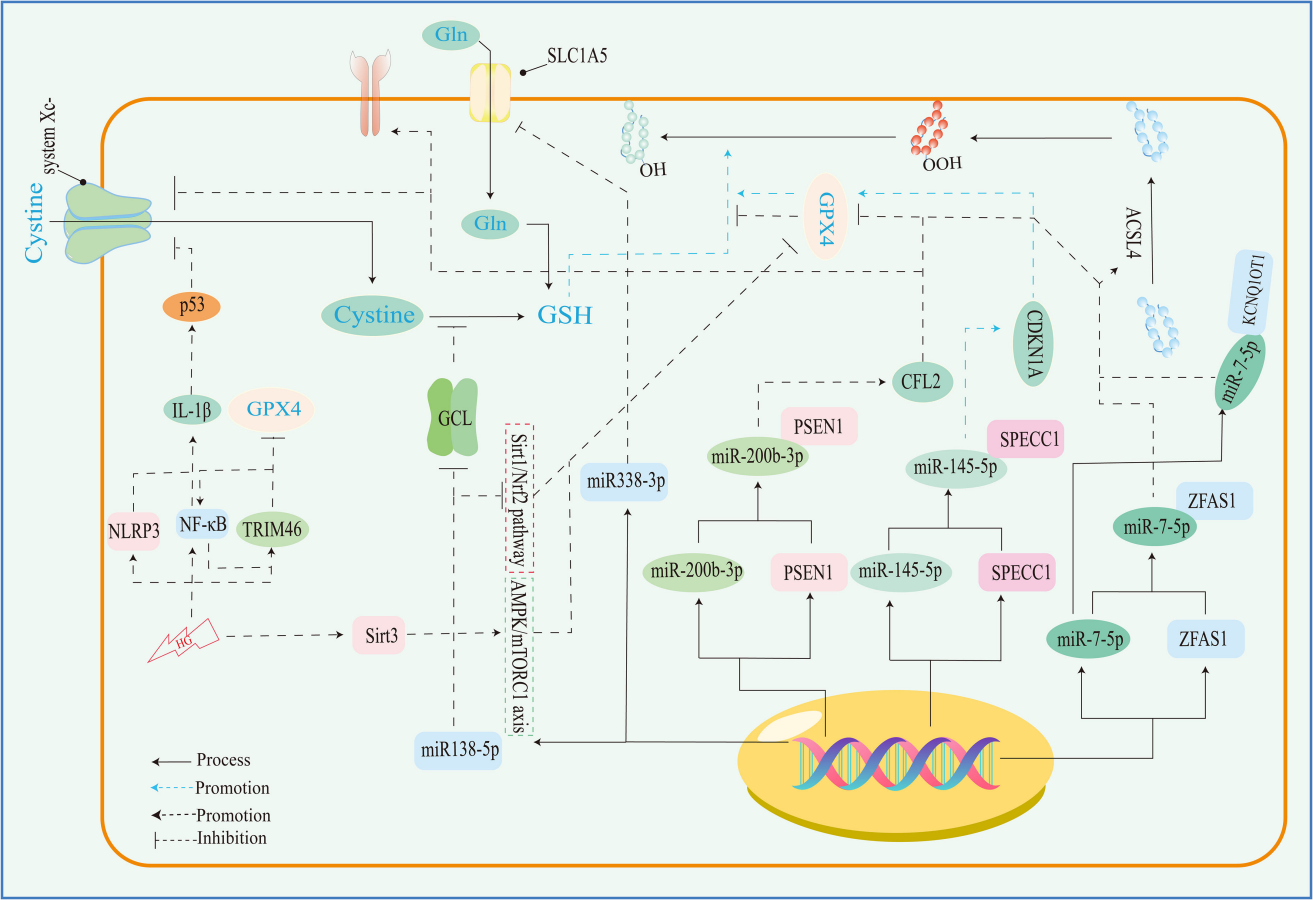
The role of ncRNA and inflammation in the resistance system of ferroptosis. sirt3, sirtuin 3; TRIM46, tripartite motif-containing 46; GCL, glutamate cysteine ligase; SLC1A5, solute carrier family A1 member 5; NLRP, NOD-like receptor thermal protein domain associated protein 3; NF-κB, nuclear factor kappa-B.

Intriguingly, despite their indirect involvement in protein translation, noncoding RNAs (ncRNAs) play a pivotal role in a diverse array of pathological and physiological metabolic processes as well as cellular states. Deftly segregated into the echelons of housekeeping and regulatory ncRNAs, these molecular orchestrators exhibit fervent activity within the central nervous system, where their nuanced fluctuations play a pivotal symphony that resonates crucially for retinal function (102, 103). Expanding upon this notion, an array of studies has unveiled the profound interplay between regulatory ncRNAs and ferroptosis within the context of DR. These regulatory ncRNAs encompass diverse categories such as microRNAs (miRNAs), long noncoding RNAs (lncRNAs), and circular RNAs (circRNAs), each acting autonomously or collaboratively to exert influence over the initiation, regulation, and deterioration of ferroptosis (**Figure 5**). Through this study, we endeavor to illuminate the intricate tapestry of interactions between ncRNAs and the ferroptotic machinery in the backdrop of diabetic retinopathy. Unveiling the role of these ncRNAs as master regulators, our exploration reveals a novel dimension in the understanding of DR pathology. Furthermore, this emerging landscape offers a promising avenue for therapeutic exploration, where the manipulation of ncRNAs might offer innovative strategies to intercept the untoward cascade of events leading to ferroptosis and the consequential retinal degeneration observed in diabetic retinopathy. Meanwhile, the entwined processes of transcription, transport, and degradation of regulatory ncRNAs reverberate within the realm of ferroptosis within the context of DR. Though the bulk of present research on the liaison between ncRNAs and pathologies primarily orbits around neoplastic conditions, this very pursuit can propel ncRNAs from the laboratory into the clinic, engendering novel therapeutic avenues for DR. Delving into the intricate mechanisms underpinning the role of ncRNAs promises to unveil transformative insights.

MicroRNAs, characterized by their single-stranded conformation, manifest as diminutive RNA molecules that predominantly facilitate degradation or translation inhibition via complementarity with the 3’-untranslated region (3’-UTR) of target mRNAs, orchestrating a mode of adverse regulation (102, 103). Concurrently, long noncoding RNAs (lncRNAs) and circular RNAs (circRNAs) assume the role of molecular sponges, ensnaring microRNAs and thus detaching them from their mRNA counterparts. This phenomenon influences the intricate network of negative regulation imposed by microRNAs upon their target mRNAs (91)

The hyperglycemic milieu stands as a pivotal instigator of pathological mechanisms in DR. In an investigative endeavor, Liu et al. (72) employed RT-qPCR methodology to unveil the upregulation of long noncoding RNA ZFAS1 expression in human retinal endothelial cells (hRECs) when exposed to HG. Intriguingly, the same study identified a substantial attenuation in miR-7-5p expression consequent to ZFAS1 overexpression. Correspondingly, Zhu et al. (104) also corroborated the deleterious impact of HG on retinal pigment epithelial cells (ARPE19), substantiating augmented intracellular levels of circ-PSEN1 and cofilin-2, while concurrently observing diminished levels of miR-200b-3p. Notably, circ-PSEN1 overexpression exhibited an inhibitory influence on miR-200b-3p, further underscoring the intricate network of interactions driven by the HG environment. Although high levels of HG have been observed to induce alterations in various classes of ncRNAs, the resultant consequences arising from these atypical expressions remain consistent. These effects encompass diminished cellular activity, lowered GSH levels, accumulation of MDA, perturbed iron homeostasis, aberrant GPX4 resistance mechanism, heightened lipid peroxidation levels, and, notably, the occurrence of ferroptosis. Distinct targeted genes can initiate diverse signaling pathways. Western blotting findings (72) demonstrated the suppression of HG-induced ACSL4 overexpression by ZFAS1 silencing and ferrostatin-1. However, this effect was substantially reversed upon miR-7-5p inhibition, illustrating the regulatory role of ZFAS1 through miR-7-5p sequestration in the modulation of ACSL4. Thus, the ZFAS1/miR-7-5p/ACSL4 axis emerges as a pivotal player in ferroptosis within retinal endothelial cells (RECs). Furthermore, interference with the ACSL4 degradation pathway results in its undue accumulation. Notably, extracellular glial maturation factor-β (GMFB), a neurodegenerative factor upregulated in early diabetic maculopathy, can redirect ATP6V1A from lysosomes, thereby impeding lysosomal acidification in retinal pigment epithelial cells (RPECs). This disruption leads to augmented ACSL4 protein levels, consequently triggering ferroptosis (100).

Recent investigations (104) have revealed that deficiency in miR-200b-3p results in heightened levels of TFRs, consequently fostering augmented iron uptake and lipid ROS generation. Moreover, following Si-circ-PSEN1 transfection, substantial elevations were observed in cellular GSH concentration, along with notable enhancements in mRNA and protein expressions of GPX4 and SLC7A11, coupled with a reduction in TFR1 expression within ARPE19 cells. However, these effects were counteracted upon augmentation of CFL2 (104). Thus, Circ-PSEN1 operates as a miR-200b-3p sponge, while cofilin-2 represents a direct target of miR-200b-3p. Remarkably, under hyperglycemic conditions, the circ-PSEN1/miR-200b-3p/cofilin-2 axis governs iron homeostasis and the resistance system against ferroptosis in RPECs (104). Furthermore, it has been documented (91) that silencing of circSPECC1 leads to diminished GPX4 protein expression, accompanied by increased accumulations of ROS, MDA, and lipid peroxide. This perturbation additionally disrupts lipid metabolism, ultimately culminating in the induction of ferroptosis. Notably, abatement of miR-145-5p expression mitigates these alterations (91). Simultaneously, the authors observed RPE atrophy and diminished retinal thickness in mice, as assessed through OCT, subsequent to subretinal administration of circSPECC1-siRNA-3 (91). Their findings propose that circSPECC1 functions as a sponge for miR-145-5p, thereby constraining its functionality and disrupting its interaction with the cell cycle protein-dependent kinase inhibitor 1 gene. This mechanism, in turn, confers safeguarding effects upon the RPE against oxidative stress-induced damage (91). Beyond the aforementioned microRNAs, it has been demonstrated that HG conditions exert a suppressive effect on the expression of alanine-serine-cysteine transporter 2 (ASCT2), a vital glutamine transporter belonging to the solute carrier family A1 member 5 (SLC1A5), by stimulating miR-338-3p transcription (97). Of noteworthy significance, ASCT2 stands as the exclusive transporter facilitating glutamine influx into cells, predominantly operating in a Na^+^-dependent manner. The downregulation of ASCT2 expression engenders a reduction in cellular proliferative activity and enhances susceptibility to oxidative stress, thereby inciting ferroptosis within the RPE.

Silent information regulator 1 (SIRT1), a class III histone deacetylase dependent on nicotinamide adenine dinucleotide, orchestrates the modulation of pertinent genes by influencing histone acetylation within the nucleus. Meanwhile, nuclear factor erythroid 2-related factor 2 (Nrf2), a fundamental basic leucine zipper (bZIP)–associated transcription factor, binds to the antioxidant response element (ARE). This binding event, transpiring under oxidative stress conditions, regulates the expression of relevant genes, thereby imparting antioxidant and anti-inflammatory effects vital for the preservation of cellular equilibrium (105). An array of investigations has underscored the pivotal role played by the Sirt1/Nrf2 signaling pathway in addressing cellular oxidative stress and inflammatory conditions. Activation of SIRT1 engenders elevated Nrf2 expression, subsequently targeting genes pertinent to cellular defense for precise regulation of their expression. In a significant study, Tang et al. (5) illuminated the inhibitory influence of miR-138-5p on the Sirt1/Nrf2 pathway. The inhibition, in turn, led to a reduction in the expression of GPX4, glutamate-cysteine ligase modifier subunit (GCLM), and glutamate-cysteine ligase catalytic subunit (GCLC), thereby exacerbating HG-induced ferroptosis within ARPE-19 cells (5).

Circular RNAs (circRNAs), characterized by their covalently closed loop-based architecture, constitute a subset of endogenous non-coding RNAs generated through back-splicing of host genes (103). This unique structure renders circRNAs devoid of free termini, rendering them resistant to degradation by nucleic acid exonucleases, thereby conferring remarkable structural stability. Among their diverse functions, a predominant mode of action entails their role as miRNA sponges. While investigations into circRNA functionality are abundant, comparatively less attention has been dedicated to comprehending their involvement in nuclear export mechanisms. Notably, Chen et al. (91) have demonstrated that the nuclear export of circSPECC1 is influenced by methylation at the m6A site. Intriguingly, demethylation events inhibit circSPECC1’s nuclear export without affecting its expression levels. Furthermore, methylation modifications at the m6A site enhance the stability of long non-coding RNA (lncRNA) KCNQ1OT1, which in turn strongly suppresses the activity of miR-7-5p (105).

### 5.2 MD and ferroptosis

Mitochondria, recognized as the cellular “powerhouses,” represent dynamic organelles possessing a dual-layer membrane structure within eukaryotic cells. Functioning as the primary locale for both cellular respiration and iron utilization, mitochondria establish an intricate network through continual processes of movement, fusion, fission, and reconfiguration (13). Mitochondria play a pivotal role in heme synthesis, being the exclusive site for its production (106). Additionally, mitochondria prominently orchestrate the biogenesis of iron-sulfur clusters (107), constituting a crucial function in cellular iron metabolism. Relative to the cytoplasm, mitochondria harbor a higher iron content (108). This iron exists within mitochondria in diverse forms, encompassing metal ions, iron-sulfur clusters, and ferritin. To facilitate the creation of iron-sulfur clusters that support the functioning of mitochondrial proteins, mitochondria require the import of iron from the cytoplasm. In turn, mitochondria furnish the cell with heme and iron-sulfur clusters, pivotal components that aid the activities of cytoplasmic and nuclear proteins. This interconnects cellular iron homeostasis with mitochondrial iron regulation. Nevertheless, the precise intricacies of import/export pathways concerning mitochondrial iron remain insufficiently elucidated. Multiple plausible routes have been postulated (109, 110): (1) the endosomal route employs a “kiss and run” mechanism, ferrying iron into mitochondria. (2) Mitochondria directly uptake iron from the labile LIP, where it is chelated by low molecular mass molecules or ferritins. (3) Iron uptake into mitochondria can occur in conjunction with chaperone proteins. (4) Fluid-phase endocytosis is a potential route. (5) Lysosomes/vesicles might orchestrate the transfer of iron from other organelles into mitochondria. Following the transport of iron via the aforementioned pathways into mitochondria, the subsequent challenge involves navigating both the outer and inner mitochondrial membranes. Nonetheless, the underlying mechanisms diverge distinctly. Presently, the prevailing consensus attributes the primary role of facilitating iron transport across the outer mitochondrial membrane to voltage-dependent anion channels. The passage of iron across the inner mitochondrial membrane is principally facilitated by mitoferrins, notably MFRN1 and MFRN2 (111). The literature that we searched predominantly explores the impact of hyperglycemia on mitochondrial dysfunction in the context of DM/DR. This encompasses modifications in membrane potential, membrane permeability, and mitochondrial dynamics. Additionally, the literature underscores how mitochondrial dysfunction disrupts iron homeostasis within the retinal cytoplasm. Regrettably, despite the classification of disorders in mitochondrial iron metabolism as a subset of mitochondrial dysfunction, the discourse on mitochondrial iron homeostasis within the context of DM/DR remains conspicuously absent within these sources.

The maintenance of mitochondrial network homeostasis plays a pivotal role in determining the proper functioning of mitochondria. As a result, MD can lead to a multitude of pathological repercussions, encompassing oxidative stress, inflammatory responses, energy depletion, aberrant glycolipid metabolism, and perturbations in iron homeostasis, among others. Conversely, the disruption of iron homeostasis in turn exacerbates irregular mitochondrial dynamics. The influence of intracellular iron levels on mitochondria predominantly occurs through oxidative damage facilitated by the Fenton reaction. Mitochondria employ several strategies to counteract oxidative stress (76): firstly, by alleviating OS and addressing misfolded proteins via the mitochondrial unfolded protein response (mtUPR); secondly, through the elimination of impaired mitochondria by means of mitophagy; and thirdly, by sustaining a reduced state at the functional active sites of proteins through the Trx/TrxR redox system.

RPECs play a pivotal role in the transportation of these energy substrates to the photoreceptors. The disruption of RPECs function precipitates impairment in photoreceptor performance. Owing to its status as a fully differentiated cell, RPECs lack the capacity to segregate and distribute defective mitochondria to progeny cells, thus necessitating efficient and timely elimination mechanisms (89). Notably, Singh et al. documented elevated Txnip expression levels in APRE-19 cells, rMC1 cells, and 661W cells exposed to sustained hyperglycemic conditions (88). Txnip, a thioredoxin-interacting protein, acts as an impediment to the Trx/TrxR redox system’s proper functioning. Concurrently, the same authors observed the onset of oxidative stress and ferroptosis in these cells, leading to diminished ATP production and mitochondrial flux (88). Consequently, Singh et al. (88) have advanced the proposition that an elevated expression of Txnip hinders the Trx/TrxR redox system, precipitating oxidative harm to mitochondria. This disruption culminates in an imbalance within the mitophagy-lysosome axis. Similarly, the research by Han et al. (112) unveiled that the silencing of Sirt3 leads to a noteworthy reduction in the protein expression of LC3B-II and beclin1. Notably, the authors demonstrated that beclin1 phosphorylation could be mediated by AMPK, a finding that astonished the researchers due to Sirt3’s role in autophagy modulation via AMPK (112). The activation of AMPK resulting from the overexpression of Sirt3 ushers in an elevated autophagic flux, inducing lysosomal burden and diminished functionality (76, 112). This phenomenon precipitates the accumulation of impaired mitochondria, thereby setting off disruptions in iron metabolism. Furthermore, this cascade contributes to a compromised performance of the mitochondrial electron transport chain (ETC), leading to a shift from proper ATP synthesis to reactive oxygen species (ROS) generation. Subsequently, cellular energy depletion ensues, alongside the oxidation of mitochondrial membrane lipids and mtDNA (76). This intricate interplay perpetuates a self-reinforcing cycle, holding cells within its grasp. Operating behind the scenes, ferroptosis emerges as a concealed assailant, amplifying mitochondrial deterioration and abating biogenesis. Conversely, MD also fosters the progression of ferroptosis (91). The intricate web of causality defies simplicity, with factors assuming diverse roles when viewed from varied perspectives.

### 5.3 Ferroptosis resistance system

Classical ferroptosis stands as a newly identified form of RCD, hinging on the accumulation of hydroxyl radicals and escalated generation of membrane lipid peroxides. These occurrences stem from a disruption in the GPX4 system, ultimately culminating in processes such as cellular swelling, osmotic lysis, and mitochondrial dysfunction (92). It follows that the perturbation of the resistance system emerges as the central instigator of ferroptosis across diverse retinal cell types. Within the glutathione peroxidase family, GPX4 plays a singular role as the sole enzyme capable of directly quenching membrane lipid peroxides. Its antioxidative potency chiefly relies on the modulation of reduced GSH levels. The expression profile of GPX4 plays a pivotal role in dictating cell destiny in response to ferroptotic signaling. GSH, a non-protein antioxidant integral to cellular defenses, is predominantly composed of glutamate, cystine, and glycine. Notably, the uptake of cystine is facilitated by system Xc^-^, an intricate reverse transporter encompassing subunits SLC7A11 and SLC3A2. Hence, the emergence of pathological system Xc^-^ operation manifests in cysteine deprivation, setting the stage for consequential events. Cysteine deprivation serves to amplify the mitochondrial tricarboxylic acid (TCA) cycle through intensified glutamine catabolism. This phenomenon, in turn, augments mitochondrial respiration, leading to a surge in mitochondrial ROS levels (60). Thus, the system Xc^-^/GPX4 axis proves its mettle as a pivotal cellular defense mechanism. Wang et al. (19) detailed their findings, revealing noteworthy increments in ROS, lipid peroxides, MDA levels, and excessive iron accumulation within RMECs subjected to H_2_O_2_ and RSL3 treatment. These perturbations were mirrored within RMEC mitochondria, accompanied by a decline in membrane potential (MMP). This constellation of changes points unequivocally toward a single outcome: ferroptosis. This culmination is anchored in the context of exacerbated oxidative damage, characterized by diminished SLC7A11 and GPX4 levels and compounded by the pathological buildup of iron (19). Coincidentally, Shao et al. (20) uncovered analogous pathological alterations within the inner and outer granular layers of diabetic model mice, as well as in ARPE-19 cells. The findings emerging from these animal and cellular investigations collectively underscore the potential of diabetes to precipitate pathological iron accumulation within the retinal milieu. Moreover, the escalated oxidative stress levels observed in this context serve as a driving force behind ferroptosis in retinal tissue cells. However, it is essential to acknowledge that the genesis of this phenomenon stems from unchecked oxidative damage, which triggers dysregulation of the Xc^-^/GPX4 system. Furthermore, an additional study has highlighted that HG levels along with IL-1β possess the capability to restrain the expression of SLC7A11. This dual action enhances the susceptibility of human umbilical vein endothelial cells (HUVECs) to ferroptosis, a response orchestrated by p53 activation (96). Notably, iron’s participation extends beyond this, as it can also instigate the activation of the NOD-like receptor thermal protein domain-associated protein 3 (NLRP3) inflammasome signaling pathway (75). In parallel, diabetic mice demonstrate a significant elevation in the expression of NLRP3 within their retinal tissue cells, which prompts apoptosis-associated speck-like protein (ASC) to cleave pro-caspase-1 into its active caspase-1 form via CARD–CARD (caspase recruitment domains) interactions (113). This ensuing cascade then facilitates the maturation of IL-1β and IL-18 (20). In effect, the elevated glucose levels exert their disruptive influence by not only activating inflammation but also exacerbating oxidative stress, thereby perturbing the classical resistance system.

NcRNAs play a pivotal role within the framework of the resistance system, alongside inflammatory factors and ROS. A study showed that the suppression of miR-7-5p resulted in the downregulation of GPX4 expression in hRECs, a trend confirmed in retinas from diabetic mouse models (72). Consequently, GPX4 can be situated downstream within the signaling cascade of the ZFAS1/miR-7-5p axis. Simultaneously, the inhibition of miR-200b-3p through transfection led to decreased mRNA and protein levels of both GPX4 and SLC7A11 in ARPE19 cells (104). Notably, restraining miR-138-5p expression also yielded an upsurge in GPX4 expression within ARPE-19 cells (5). Likewise, the knockdown of circSPECC1 precipitated a downregulation of GPX4 protein expression in both fRPE and ARPE-19 cells (91). These findings underscore the essential regulatory roles of circSPECC1, miR-138-5p, miR-200b-3p, and miR-7-5p within the Xc^-^/GPX4 system of ARPE19 and RPECs. Moreover, it is evident that the hyperglycemic milieu governs the susceptibility of retinal tissue cells to ferroptosis, leveraging the influence of these factors. A recent study highlighted the potential of Lipocalin 2 to counteract ferroptosis by upregulating the expression of GPX4 and xCT components through the action of ETS1, a transcription factor (65). Notably, SIRT3, recognized as a classic NAD+-dependent mitochondrial protein deacetylase, serves as an activator of AMPK. In a pathophysiological context, HG levels provoke an overexpression of SIRT3 (85). Notably, Han et al. (112) uncovered, through Western blot analysis, that the silencing of SIRT3 hinders AMPK phosphorylation, thereby prompting an elevation in mTORC1 activity and GPX4 levels. Consequently, the overexpression of SIRT3 mitigates GPX4 expression by activating the AMPK-mTORC1 axis (112).

All these aforementioned factors possess the capacity to elicit or hinder the expression of constituents within the Xc^-^/GPX4 system. Notably, the breakdown of this system also holds profound significance in the orchestration of ferroptosis. Tripartite motif-containing 46 (TRIM46) is a gene situated on chromosome 1q21, encoding a protein characterized by a RING finger structural motif. This protein qualifies as a member of the E3 ubiquitin ligase family (114). Of note, the ubiquitin-proteasome system plays a role in orchestrating the ubiquitinated degradation of GPX4 during the process of ferroptosis. In diabetic patients, an elevation in mRNA levels of TRIM46 is distinctly observable (85). Fresh insight has emerged from a recent study, indicating that HG levels exert a stimulatory influence on TRIM46 mRNA transcription. This response extends to protein translation within hRCECs, presenting a concentration-dependent manner of action (12).

Upon the knockout of TRIM46, a notable amelioration is observed in the context of HG-induced escalation in membrane lipid peroxidation levels, alongside an upregulation in prostaglandin endoperoxidase synthase 2 (PTGS2). Moreover, the proliferative capacity of hRCECs experiences a decrease (85). Intriguing insights surface from the study of Zhang et al.; through co-immunoprecipitation assays, the authors unveiled that TRIM46 engages with GPX4. Notably, no discernible alteration was noted in the level of GPX4 mRNA. Instead, an elevation was witnessed in the protein levels within hRCECs subjected to TRIM46 knockdown (85). This led to the proposal that the hyperglycemic milieu could trigger the expression of TRIM46. In sequence, TRIM46 sets its sights on GPX4, instigating the ubiquitinated degradation of GPX4. This orchestration consequently blunts the resistance against ferroptosis within hRCECs. Furthering our understanding, Shen et al. (12) brought forth findings that TRIM46 is a catalyst in the ubiquitination of IκBα. This process paves the way for protease-dependent degradation within hRCECs. Subsequently, the liberation of NF-κB into the nucleus ensues, thereby catalyzing the transcription of an array of downstream inflammatory factor genes, encompassing TNF-α, IL-6, and IL-1β, among others. Worth noting is the disruptive potential of IL-1β, which targets the Xc^-^/GPX4 system through the p53-xCT-GSH axis (96). Additionally, the introduction of pyrrolidine dithiocarbamate, an inhibitor of NF-κB, substantially reduced the levels of both TRIM46 mRNA and protein (12). These observations collectively suggest the likelihood of a bidirectional promotion between the TRIM46 and NF-κB signaling pathways, with the hyperglycemic environment standing as the instigator of this intricate feedback loop.

## 6 Ferroptosis and treatment of DR

Contemporary therapeutic strategies are primarily directed toward combating microangiopathy in the context of DR. However, it is crucial to acknowledge that in cases of advanced DR, ameliorating microangiopathy does not translate to improved visual acuity (79). As previously elucidated, the pivotal roles of iron and ferroptosis in DR cannot be overstated. Consequently, an imperative emerges for the development of pharmaceutical interventions capable of rectifying retinal ferroptosis. The initiation of ferroptosis is intricately tied to the accrual of reactive iron and lipid peroxidation. To counter retinal ferroptosis, a dual-pronged strategy emerges: firstly, addressing the surplus accumulation of reactive iron within the retinal context, coupled with the restraint of membrane lipid peroxidation. Simultaneously, the mode of drug administration warrants meticulous consideration, necessitating an assessment of the merits and drawbacks associated with local versus systemic delivery. Given that the regulation of ferroptosis involves a multi-faceted process, a plethora of potential avenues can be explored to prevent and manage DR. This pursuit adheres to the aforementioned principles, thus encouraging the identification of an expanded array of targets with the potential to effectively mitigate DR.

The pathological accumulation of reactive iron acts as a pivotal trigger for ferroptosis, thus highlighting the imperative of effectively mitigating retinal iron overload in patients with DR. For this subset of patients, strategies aimed at rectifying this imbalance assume paramount significance. Several approaches can be considered, including the use of iron chelators, the augmentation of cell membrane FPN abundance, or the inhibition of TFR expression, among others. Nevertheless, conventional iron chelators are marked by substantial systemic side effects and their restricted ability to permeate the BRB, a case in point being desferrioxamine (13). As such, the quest for iron chelators capable of traversing the BRB and conferring targeted action within the retina becomes all the more pressing. Enter lactoferrin (LF), an iron-binding glycoprotein that originates from mucous cells and neutrophils. LF holds the unique capability to breach the BRB and exhibits substantial bioavailability (14). Notably, LF serves a dual function: it acts to chelate reactive iron within the retinal milieu, thereby curtailing the generation of ROS. Furthermore, LF directly scavenges ROS and exhibits the ability to downregulate the expression of TNF-α and ILs (11). These multifaceted attributes underscore the formidable potential of LF, an avenue that has surprisingly garnered less attention within the realm of DR treatment studies. Simultaneously, research conducted by Moos (13) et al. unveiled a promising avenue. A novel synthetic derivative of α-lipoic acid, known as PMX500FI, stands out for its capacity to sequester iron within the retina while effectively crossing the BRB. This compound additionally activates the Nrf2 signaling pathway, suppressing oxidative stress. Significantly, PMX500FI demonstrates the added benefit of ameliorating mitochondrial function. Moreover, it orchestrates a reduction in iron uptake and lipid peroxide production, thereby triggering the activation of the miR-200b-3p/TFR axis (115).

Suppression of lipid peroxidation remains a paramount objective. This endeavor aligns with the broader goal of mitigating disruption within the ferroptosis resistance system. Contemporary investigations underscore the potency of strategies such as elabela and ferrostatin-1 in alleviating retinal ferroptosis. A wealth of both cellular and animal experiments substantiates the efficacy of elabela and ferrostatin-1 in tempering ferroptotic processes within the retina, achieved by activating the XCT-GPX4 axis (19, 20). Intriguingly, ferrostatin-1 presents additional mechanisms beyond the elevation of GPX4 expression to thwart the accrual of lipid peroxidation products; however, the precise intricacies of these mechanisms remain to be elucidated (72). Furthermore, Tang et al. (5) have contributed noteworthy insights. Their findings elucidate how astragaloside-IV (AS-IV) effectively suppresses miR-138-5p expression, thereby engendering heightened Sirt1/Nrf2 activity. This cascade of events culminates in the upregulation of GPX4, consequently reducing lipid peroxidation levels within the retinal milieu. Beyond the facets highlighted earlier, the signaling pathways expounded upon within the ferroptosis and DR domain emerge as promising targets for the innovative treatment of DR. A case in point involves the strategic attenuation of the p53/xCT/GSH axis and the miR-338-3p/SLC1A5 axis, a maneuver that elevates intracellular GSH levels, thereby mitigating susceptibility to ferroptosis (96, 105). Furthermore, interventions targeting the circ-PSEN1/miR-200b-3p/cofilin-2 and miR-138-5p/Sirt1/Nrf2 pathways hold the potential to bolster GPX4 activity (5, 104). This augmentation fortifies cellular resilience against ferroptosis by invigorating the protective cellular defense apparatus. In contrast, manipulation of the lncRNA ZFAS1/miR-7-5p axis engenders a notable shift. This maneuver culminates in the downregulation of ACSL4 expression (72), resulting in the diminishment of a steadfast enabler of ferroptosis. In analogy, it is akin to removing the fuel that sustains the fire beneath the crucible.

The amelioration of DR progression is also closely tied to the inhibition of ER stress (78). This inhibition is associated with the diminution of cellular oxidative stress and inflammation levels, the enhancement of intracellular iron homeostasis, and the attenuation of membrane lipid peroxidation. Wang (116) et al. have conducted research illuminating the potential of blueberry anthocyanin extracts in quelling ER stress through the miR-182-8/oxoguanine-DNA glycosylase (OGG1) axis, thus offering relief for DR patients. Moreover, the compound lactucaxanthin showcases analogous attributes by thwarting ER stress and curtailing the expression of inflammatory factors. This intervention extends its efficacy to reducing lipid peroxidation, thereby interrupting the intricate interplay between oxidative stress, ER stress, and inflammation (117).

Despite the current scarcity of effective and accessible therapeutic agents, the realm of drug-like molecules spans a vast expanse within the chemical spectrum. Within this context, the potential for intervention is as boundless as the ocean. Employing a repertoire of strategies, we can delve into the realm of virtual screening. Utilizing techniques such as molecular docking and molecular dynamics simulations, we can identify compounds or molecular entities with the capacity to interact with the active components of the aforementioned signaling pathways. This approach, grounded in computer-based drug design, sets the stage for scrutinizing drug candidates. Subsequently, we can translate these findings into cellular assays, effectively gauging the biological activity and potential ferroptosis-inhibitory roles of the screened compounds or molecular assemblies. This integrated approach empowers us to harness the computational prowess of computers to sift through existing molecular libraries, expediting the identification of viable therapeutic molecules or drug groups. Intriguingly, a noteworthy discovery by Stynen et al. (118) has illuminated the ability of metformin to intervene in intracellular iron homeostasis, inducing a state of “iron starvation” within cells. Unfortunately, no investigations thus far have pointed toward a similar effect within the human retina. However, liraglutide, a prominent antihyperglycemic agent, stands out as a beacon of hope. It exerts its influence by restraining oxidative stress and ER stress. This intervention is achieved through the augmentation of NrF2 and Trx expression, orchestrating a deceleration in the progress of DR through the attenuation of ferroptosis (119). The ramifications of retinal iron overload extend beyond the realm of severe ferroptosis. This phenomenon incites the elevation of renin expression, thus kindling the activation of the retinal renin-angiotensin system (RAS) via succinate-GPR91 signaling within retinal pigment epithelial (RPE) cells. This cascade, in turn, intensifies retinal microangiopathy and compromises the integrity of the blood-retinal barrier (BRB) architecture (75). In line with this, a cross-sectional cohort study of considerable scale reported substantially elevated serum renin levels in patients with type 1 diabetes and proliferative DR (PDR) compared to those with non-proliferative DR (NPDR) (P=0.044) (120). This substantiates the notion that iron overload could potentially exacerbate DR progression via heightened renin expression. Further insights emerge from a study (121) that demonstrates angiotensin-converting enzyme (ACE) exacerbates BRB disruption during DR by activating the transforming growth factor-beta 1 (TGF-β1)/Smad signaling pathway. With this in mind, the application of ACE inhibitors (ACEIs) or angiotensin receptor blockers (ARBs) emerges as a theoretically sound strategy to curb the retinal RAS system. This approach holds promise in mitigating the microangiopathic manifestations of DR to a certain extent (75). In light of these discoveries, the landscape of therapeutic avenues for DR remains exceptionally promising. The prospect of simultaneously ameliorating hyperglycemia while concurrently alleviating retinal ferroptosis looms large, igniting the quest for innovative solutions in the battle against DR.

## 7 Discussion

**Figure 6:**
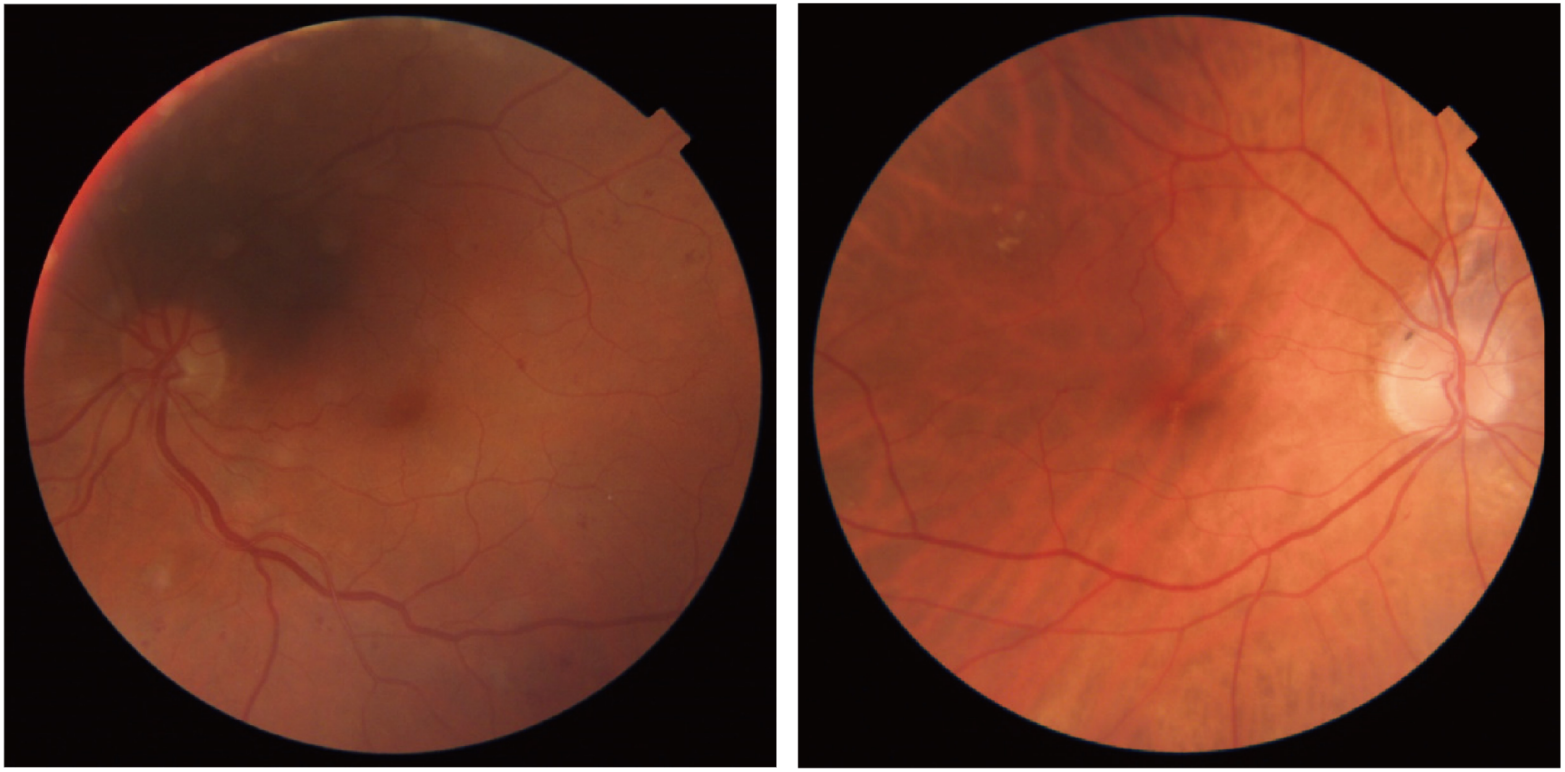
Dilation-free funduscopies of DR from (122). The left image shows multiple small hemorrhages and microaneurysms. The right image shows peripapillary pigment, hard exudates in peripheral macula, and more subtle exudates near the fovea.

The symptoms accompanying the initial stages of DR, such as visual blurring, can often be misconstrued as presbyopia, particularly among elderly diabetic patients (**Figure 6**). Regrettably, if these early indicators are overlooked, the condition may progress to the point of retinal detachment (RD). This dire circumstance leaves treatment options confined to surgical intervention, inevitably heightening the risk of irreversible vision impairment and blindness. Presently, the approach of anti-VEGF therapy stands out as notably efficacious in managing central macular edema and PDR (123). This therapeutic avenue demonstrates comparable effectiveness to pan-retinal photocoagulation over a span of 5 years (124). Agents such as ranibizumab, aflibercept, and conbercept play pivotal roles within this framework (125, 126). While its therapeutic efficacy is compelling, it exclusively targets one among several risk factors. Diabetic retinopathy’s emergence and progression stem from a complex interplay of oxidative stress, ER stress, inflammatory responses, and ferroptosis. Intriguingly, iron overload exacerbates these pathological transformations while also inciting retinal VEGF release, facilitated by the succinate/GPR91 pathway. Thus, the management of retinal iron overload assumes paramount importance. The foregoing overview highlights a multitude of factors contributing to abnormal iron accumulation within the retina. Broadly, these factors can be categorized into two groups: systemic factors, encompassing age, metabolic disorders, oxidative stress, MD, and inflammatory cascades, and local factors, including structural breaches of the BRB and impediments to retinal FNP. Beyond the age factor, the pathological buildup of iron amplifies the deleterious effects associated with these other multifaceted contributors. Consequently, addressing the pivotal role of “iron” as the nexus of these intricate relationships becomes imperative.

In overviewing the aforementioned studies, there emerge five pivotal dimensions of iron overload, collectively illuminating its intricate mechanisms underlying retinal damage. Firstly, iron overload’s detrimental impact on the retina is rooted in its capacity to induce oxidative stress through the Fenton reaction, triggering a cascade of secondary consequences such as MD, ER stress, and inflammatory responses. Secondly, the intricate orchestration of intracellular iron homeostasis intertwines with the realm of inflammatory factors, hence iron overload’s potential to incite an onslaught of inflammatory mediators. Thirdly, iron’s involvement in glycolipid metabolism renders the retina susceptible to metabolic dysregulation in the context of iron overload. Fourthly, iron overload intricately mediates the activation of retinal RAS and VEGF, thereby exacerbating retinal microvascular damage. Lastly, iron overload progression culminates in ferroptosis, a form of regulated cell death driven by membrane lipid peroxidation. Consequently, in probing iron overload or “ferroptosis” as targets for DR therapies, a holistic perspective must be upheld, considering the interconnectedness of these processes, either synergistically or antagonistically. It is pertinent to note that while the body of research outlined herein is mainly anchored in cellular and animal experimentation, there remains a need for further exploration in therapeutic investigations targeting iron overload and ferroptosis.

## Author contributions

JO collected and reviewed the literature and wrote the manuscript. LZ helped with the revision and design of the manuscript and collection and analysis of the literature. QW read and approved the content of the manuscript. All authors contributed to the article and approved the submitted version.
